# Expanding Application of Optical Coherence Tomography Beyond the Clinic: A Narrative Review

**DOI:** 10.3390/diagnostics15091140

**Published:** 2025-04-29

**Authors:** Tutut Nurjanah, Milin Patel, Jessica Mar, David Holden, Spencer C. Barrett, Nicolas A. Yannuzzi

**Affiliations:** 1 Department of Ophthalmology, Bascom Palmer Eye Institute, University of Miami Miller School of Medicine, Miami, FL 33136, USA; 2 Fakultas Kedokteran, Universitas Yarsi, Jakarta 10510, Indonesia; 3 Philadelphia College of Osteopathic Medicine, Suwanee, GA 30024, USA

**Keywords:** optical coherence tomography (OCT), age-related macular degeneration (AMD), dry AMD, wet AMD, home OCT, intraoperative OCT, vitreomacular traction (VMT), pars plana vitrectomy (PPV), screening, mobile OCT, future of ophthalmology, medical imaging

## Abstract

Since its introduction, optical coherence tomography (OCT) has significantly progressed in addressing its limitations. By integrating artificial intelligence and multimodal imaging, OCT enhances both speed and image quality while reducing its size. OCT continues to advance, offering new possibilities beyond the in-office setting, including intraoperative applications. This review will explore the different types of home OCT and intraoperative OCT, as well as the uses of each device and their future potential in ophthalmology.

## 1. Introduction: OCT in Home and Intraoperative Settings

OCT is a non-invasive imaging technology that utilizes light waves to produce high-resolution, cross-sectional images of retinal layers and ocular structures. OCT allows for precise in vivo assessment of retinal morphology and pathology by measuring the time delay and intensity of reflected light. Ophthalmic imaging has come a long way since a team of researchers led by Dr. James Fujimoto at the Massachusetts Institute of Technology (MIT) and assisted by ophthalmologists Joel Schuman, M.D., David Huang, M.D. Ph.D., and Carmen Puliafito, M.D., began exploring low-coherence interferometry to image biological tissues in the 1980s. Later, in 1991, David Huang developed OCT in the Fujimoto lab, and the first in vivo retinal images were published in 1993 [1].

Early time-domain OCT (TD-OCT) systems, introduced clinically in 1997, used a moving reference mirror to detect reflected light, offering axial resolutions of 10–15 µm but limited by slow acquisition speeds [2,3]. The emergence of spectral-domain OCT (SD-OCT) in the mid-2000s marked a major leap, replacing the moving mirror with a spectrometer and enabling higher speed (thousands of A-scans/seconds) and improved axial resolution (5–7 µm) [4]. More recently, swept-source OCT (SS-OCT) has advanced the field further, utilizing a tunable laser for deeper tissue penetration, enhanced imaging through media opacities, and faster scan rates [5]. Additionally, the advent of OCT angiography (OCT-A) in 2014 allowed for noninvasive visualization of retinal and choroidal vasculature without the need for dye injection. It is used to identify perfusion defects, detect microaneurysms, and assess neovascularization [6].

OCT has become a mainstay in fast, non-invasive diagnoses of both anterior and posterior segment pathology and has become instrumental in managing ophthalmic diseases and assisting in surgical planning. SD-OCT enables the precise characterization of retinal diseases by providing detailed cross-sectional imaging and allowing for the assessment of lesion size, reflectivity, and location. SD-OCT has become the primary method of monitoring AMD, where drusen are identified as RPE elevations and classified by composition and size into hard, cuticular, pseudodrusen, or soft drusen [7]. Often associated with AMD, pigment epithelial detachment (PED) can also be further characterized on OCT as drusenoid, fibrovascular, or serous [7,8]. In late-stage non-neovascular AMD, OCT is used in conjunction with fundus autofluorescence to quantify and evaluate the progression of Geographic atrophy (GA) [9,10].

Despite the vast clinical utility of OCT, practical limitations of the modality, such as its limited accessibility, limited portability, technical skill required for acquisition, and the occasional need for pupil dilation, have necessitated further innovation [11]. The development of home-based, handheld, and intraoperative OCT has expanded the possibilities of retinal imaging beyond traditional clinic-based systems. These technologies offer high-resolution imaging in settings and patient populations where conventional OCT is limited, particularly in pediatrics. Intraoperative OCT supports surgical decision-making and real-time assessment [12], while handheld OCT allows imaging in non-cooperative or bedside settings [13].

This review synthesizes the current landscape of applications of OCT outside traditional clinical settings, namely at-home and intraoperative OCT.

## 2. Home OCT

### 2.1. Home OCT for Early Detection

Home-based OCT, a new portable technology, is an exciting frontier in the early detection and monitoring of ocular pathology. Currently, home-based OCT devices facilitate adjustments during office visits, alleviating the in-office time burden on patients. In a prospective observational study by Liu et al. and a systematic review by Dolar-Szczesny et al., it was found that home OCT demonstrated excellent sensitivity and specificity compared to in-person assessments [14,15].

One of the primary conditions benefiting from home OCT is age-related macular degeneration (AMD), a leading cause of irreversible vision loss in people over 50, with prevalence expected to increase significantly by 2030 [16,17,18,19]. However, early detection is often delayed because the progression of this disease tends to be asymptomatic [20]. AMD is categorized into early, intermediate, and late stages [21]. AMD can lead to blindness due to geographic atrophy, neovascular AMD (nAMD), or a combination of both. The exudative form is more rapidly progressing than the atrophic form, affecting 10% to 15% of patients but accounting for 90% of acute blindness cases [22,23,24].

The diagnosis of AMD involves various visual tests and multimodal imaging, which have greatly improved in recent years. These advancements in imaging techniques have transformed the field, enabling a more systematic method for detecting and classifying AMD in its early stages. One of the most common and user-friendly tests is the Amsler grid. This simple tool featuring a grid pattern is designed to identify scotomas or metamorphopsia and demonstrates reliability in detecting visual distortions by measuring grid perception that may appear wavy, irregular, or distorted [25,26]. While it remains widely used due to its convenience, the Amsler grid suffers from **low sensitivity** in detecting progression from dry to wet AMD and is limited by **subjective patient reporting** and **poor compliance**. In contrast to many ophthalmic tests, the Amsler grid relies more heavily on subjective patient reporting and at-home use, while other tests provide objective measurements or are performed in the office under supervision. These characteristics likely contribute to the grid’s low sensitivity testing [27]. In contrast, modern imaging technologies such as color fundus photography, OCT, infrared reflectance (IR), fluorescein angiography, indocyanine green angiography, and fundus autofluorescence (FAF) provide objective, detailed assessments of structural changes in the retina [28,29,30].

In addition to imaging advancements, a growing body of research is shedding light on AMD progression. Pooled data analyses of three prospective population-based cohorts conducted by Joachim et al. indicated that for unilateral AMD cases, the risk of developing AMD in the other eye within five years is 19–28% for any AMD cases and 27–68% for unilateral late AMD cases [31]. However, understanding the various factors contributing to the progression of AMD remains incomplete. Lifestyle choices, such as smoking, body mass index (BMI), and diet, significantly affect disease progression [32,33]. Due to the progressive nature of AMD, researchers are seeking reliable biomarkers for AMD, such as drusen volume, pigment changes, and early signs of atrophy [34]. Among these, contrast sensitivity seems to be one of the most promising early indicators. Furthermore, the use of AI has seemingly brought us closer to understanding the progression to late AMD and its severity [35] and the transition from intermediate AMD to GA by cross-validating multiple images and tracking the changes across different types of equipment [36].

Beyond AMD, other retinal conditions such as diabetic macular edema (DME) also stand to benefit significantly from early detection through home OCT. DME is a major contributor to vision loss at any stage of diabetic retinopathy. It involves the accumulation of fluid in the macula due to a compromised blood-retinal barrier [37]. Like AMD, DME often progresses silently, with significant damage occurring before symptoms are noticed [38]. Advanced stages of DME may be present even in patients who are not experiencing visual symptoms. Since DME is often asymptomatic in its early stages, early detection through regular monitoring is essential to ensure early detection and more frequent monitoring, possibly leading to improved long-term control. However, screening diabetic patients for eye problems can be difficult because it requires extensive exams, pupil dilation, advanced imaging, and timely referrals to eye specialists [39]. For type 1 diabetes, even in patients without evidence of diabetic retinopathy, screening should begin five years after diagnosis, while those with type 2 diabetes should be screened promptly at diagnosis and annually thereafter. Early treatments are essential in preserving the vision [40].

Another condition that emphasizes the importance of accessible imaging is Retinal vein occlusion (RVO), another leading cause of vision loss, alongside AMD and DME, in individuals over 55 years old. Major risk factors include advancing age, cigarette smoking, diabetes, and elevated diastolic blood pressure [41,42]. In all three diseases, AMD, DME, and RVO, biomarkers identified through OCT, such as central retinal thickness, intra retinal fluid (IRF), subretinal fluid (SRF), and PED, serve as key indicators of disease activity [43,44], making OCT important for the early detection and monitoring of disease.

#### Type of Home OCT Used, Resolution, and Image Quality

Currently, the only commercially available home-based OCT device is the ForeseeHome OCT by Notal Vision. While others are still waiting for more reliable data before being approved and released by the Food and Drug Administration (FDA) (Table 1) [45]. In this section, we will review those devices currently approved or still undergoing clinical investigation.

1. The ForeseeHome Device

**Figure 1:**
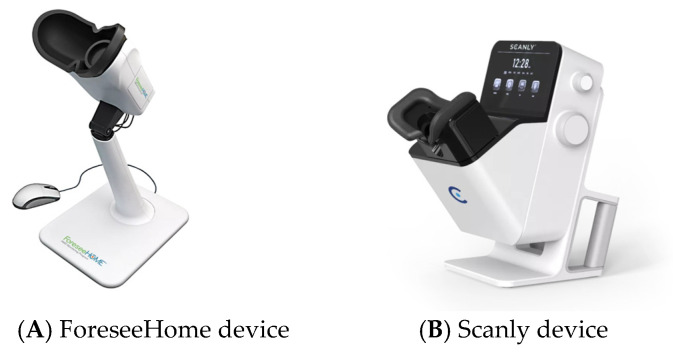
Courtesy of Notal Vision Company(Mannasas, Virginia).

The ForeseeHome (Figure 1A) is the first FDA-approved home monitoring device, a home-based imaging system that is used daily for 3 min per eye to monitor vision changes that may indicate the development of Choroidal neovascularization (CNV). It employs a patented preferential hyperacuity perimetry (PHP) technology to identify visual changes, such as metamorphopsia or scotoma, that can signal recent-onset CNV resulting from AMD. After establishing a baseline, it alerts the patient and physician to schedule necessary in-office evaluations for early intervention if any changes are detected. The ForeseeHome device uses “preferential looking”, which works by detecting changes in how a patient sees straight lines. When a patient notices a bend or distortion in the line, the device can identify where the problem is and how it has occurred. Research indicates that the device accurately identifies signs of neovascular AMD over 80% of the time, significantly surpassing rudimentary methods such as the Amsler grid. A randomized study by the AREDS2-HOME Research Group compared home monitoring plus standard care with standard care alone in patients at high risk of developing CNV. The ForeseeHome device was found to be effective, detecting CNV as progression to wet AMD earlier than standard care. The HOME trial showed that 87% of patients using the device maintained vision of 20/40 or better, compared to 62% in the standard care group. In addition, the device issued a system alert for 52% of conversions before they occurred and could detect changes in vision at the same visual acuity, even if the patient did not notice them. Their internet-based system also allows retina specialists to track patient usage and ensure consistent monitoring. A study by Loewenstein et al. further confirmed the device’s accuracy in detecting early-stage CNV and intermediate AMD [46,47,48].

2. The Notal Vision Home (NVHO) “SCANLY”

**Figure 2:**
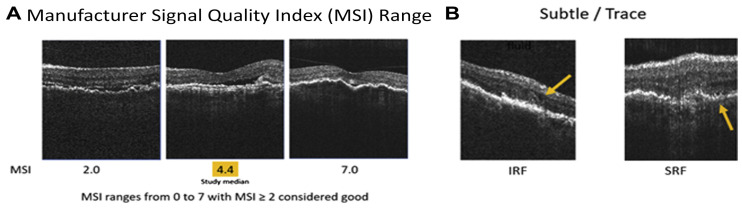
Home OCT images acquired using the Notal Vision device. (**A**), Representative B-scan images illustrating a range of manufacturer signal quality index (MSI) levels. (**B**), B-scans identified by investigators showing minimal fluid presence (indicated by arrows), as well as intraretinal and subretinal fluid. The MSI serves as an objective metric to assess the quality of the images, with a score of 2 or higher generally considered acceptable. IRF = intraretinal fluid; MSI = manufacturer signal quality index; SRF = subretinal fluid. Image from Liu et al. Prospective, Longitudinal study: Daily Self-Imaging with Home OCT for Neovascular Age Related Macular Degeneration [14].

The Scanly/NVHO system is a remote imaging device designed for individual use outside of clinical settings, utilizing a specialized SD-OCT scanner (Figure 1B). Integrated with the Notal OCT Analyzer (NOA), an artificial intelligence-based software, it enables fully automated detection and quantification of retinal fluid, including IRF and SRF. The system begins with a one-time automated calibration procedure that adjusts to the user’s specific refractive error and axial length to ensure accurate imaging. The NVHO performs a scan using a horizontal raster of 88 B-scans over a 3 × 3 mm area (10° × 10° field of view) centered on the eye’s point of fixation. Following each self-imaging session, the data are automatically transmitted to the Notal Health Cloud via an integrated cellular modem. From these raw data, cube scans are reconstructed and made available for remote review by healthcare professionals through a web-based viewer. The NOA also processes the scans to detect and quantify retinal fluid, generating annotated B-scans that highlight fluid areas, en-face maps of fluid thickness (separately for IRF and SRF), and a ranked order of B-scans based on fluid area [14,49,50]. Additionally, the NOA provides a ranked list of B-scans based on the extent of fluid detected. To assess image quality, the NVHO device calculates the Manufacturer Signal Quality Index (MSI), which offers an objective and quantifiable measure of scan quality (Figure 2) [49].

3. Self-Examination Low-Cost Full Field OCT (SELFF OCT)

**Figure 3:**
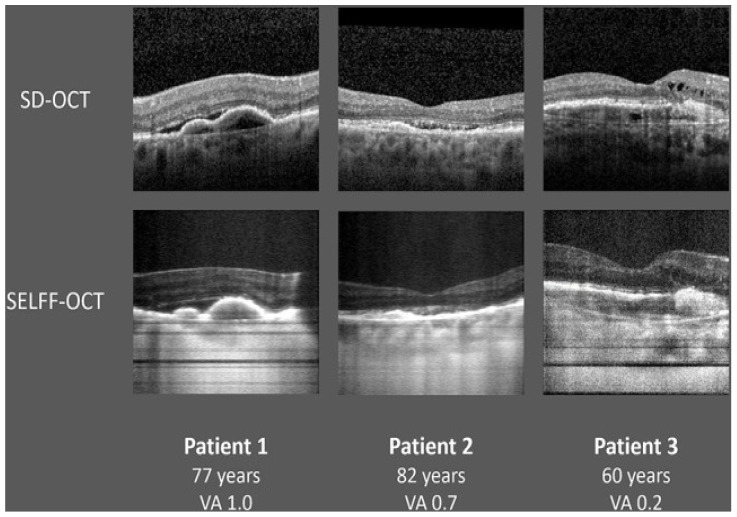
Comparison of exemplary SELFF-OCT scans with a representative SD-OCT scan. Both imaging modalities demonstrate the same retinal structures; however, the SELFF-OCT images have a smaller field of view and exhibit a lower signal-to-noise ratio (SNR) compared to the SD-OCT scan. Foveal B-scans from three representative patients acquired with spectral-domain OCT (SD-OCT, **top**) and self-examination low-cost full-field OCT (SELFF-OCT, **bottom**). Subretinal fluid (SRF) is visible in patients 1 and 2, intraretinal fluid (IRF) in patient 3, and pigment epithelium detachment (PED) in all cases. Despite a smaller field of view and lower signal-to-noise ratio (SNR) in SELFF-OCT, key AMD biomarkers (SRF, IRF, and PED) are clearly detectable and comparable to SD-OCT. Picture from von der Burchard et al. Self-Examination Low-Cost Full-Field Optical Coherence Tomography (SELFF-OCT) for Neovascular Age-Related Macular Degeneration: A Cross-Sectional Diagnostic Accuracy Study [51].

SELFF-OCT is designed for patient self-examination and has been shown to be independently operable by patients, including those over 50. The SELFF-OCT system uses a low beam to scan the retina. It shines a low-power light at a wavelength of 840 nanometers and captures a 3D image of a small area of the retina, about 4.5 by 1.4 mm. The scan can show fine details, with a depth resolution of about 12 μm and overall image sharpness of around 17 μm, while a complementary metal-oxide semiconductor (CMOS) camera captures the reflected light. This light interferes with a reference beam to create patterns at specific retinal depths, which are then processed into images. By adjusting the reference arm, the entire retina can be scanned in under one second. The system first conducts a low-resolution overview scan to locate the retinal pigment epithelium (RPE), followed by detailed high-resolution scans around the RPE, capturing a clear 3D view of the retina (Figure 3) [50,51]. This device is intended to be cheaper and more comfortable to use by the elderly by accommodating three 3D-printed headrests that allow for more stable and rigid head positioning. Compared to the first prototype, the optical setup was optimized to minimize reflections and provide more consistent illumination of the retina. Additionally, the system now corrects for defocus and astigmatism numerically during processing, eliminating the need for manual diopter adjustments [51].

4. Sparse OCT

**Figure 4:**
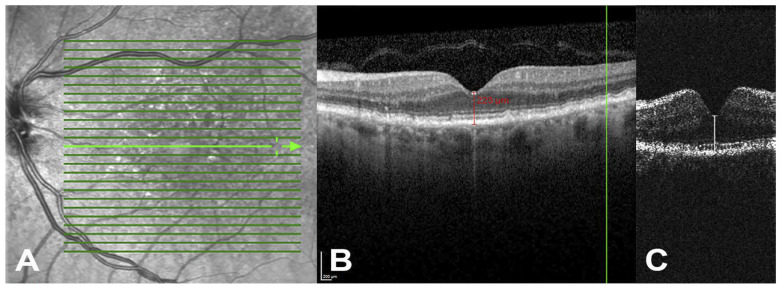
Comparison between the standard Heidelberg Spectralis OCT and the new investigational spOCT system. (**A**) Scanning laser ophthalmoscopy (SLO) image of an eye with AMD. (**B**) Cross-sectional image from the Spectralis showing manual central retinal thickness (CRT) measurement, and (**C**) the corresponding CRT measurement using spOCT. Green horizontal lines indicate the individual B scan slices acquired across the retina. Each line corresponds to a cross-sectional scan. The vertical arrow with double heads, this point to specific slice shown in panel B. The spOCT uses lower resolution to allow faster and repeated scans. Despite the reduced image detail, CRT measurements from the spOCT were similar to those from the standard device. Image from Maloca et al. Safety and Feasibility of a Novel Sparse Optical Coherence Tomography Device for Patient-Delivered Retina Home Monitoring [54].

Sparse OCT (spOCT) was developed using a method called Compressed Sensing (CS) with SD-OCT. This method allows for fewer data points to be collected while still creating accurate images. Instead of needing a large amount of data for processing, spOCT uses just a small sample of information, which makes the imaging process faster and more efficient (Figure 4) [52,53,54]. Prototype sparse OCT devices, such as MIMO_02, have been designed for potential use in home settings. Unlike conventional commercial SD-OCT systems, this device operates with a lower scanning density. They capture images over an area of 3.8 × 3.8 mm with pixel resolutions ranging from 50 × 50 to 150 × 150, and achieve a depth resolution of 2048 pixels across a depth of 4.2 mm [54].

5. SmartOCT

**Figure 5:**
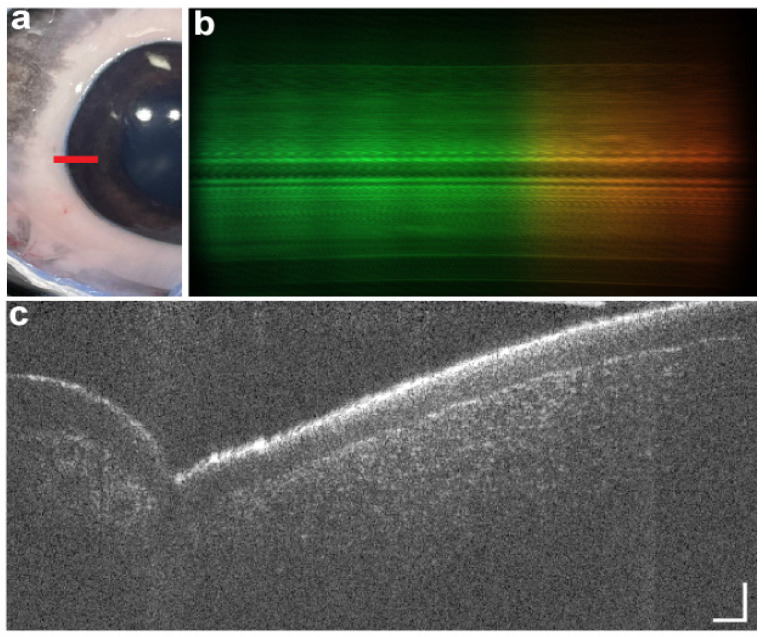
Sample image of ex vivo porcine anterior segment. Photograph of the anterior segment of the eye (**a**) with the red line showing the location of the B-scan. Raw spectrum (**b**) and 10-frame averaged B-scan (**c**) of the corneal limbus. Scale bars are 150 µm along the y-axis (horizontal) and 50 µm along the z-axis (vertical). Image from Malone et al. SmartOCT: Smartphone-Integrated Optical Coherence Tomography [55].

SmartOCT is the first system that uses a smartphone’s built-in features to generate images of the retina. It is designed using a line-field OCT (LF-OCT) setup, which allows it to capture 2D cross-sectional images and enable single-shot B-scan imaging, making it simpler and faster. To reduce the distortion, this system uses smartphone lenses to direct the light into the smartphone sensor (Figure 5). To prevent any movement and misalignment, the device is mounted securely to a 3D-printed holder [55].

**Table 1:** Type of home-based OCT.

Device Name	FDA Approved	Commercialized	System Type	Purpose	References
ForeseeHome OCT by Notal Vision	Yes	Yes	Preferential Hyperacuity Perimetry (PHP)	To enable earlier detection of Wet AMD by notifying the physician when changes are detected.	Chew EY et al. 2014 [48]
Scanly by Notal Vision	Yes	No	SD OCT with AI integrated with Notal OCT Analyzer (NOA) an AI analyzer	Self-operated tele-connected device for daily imaging between office visits	Mathai et al. 2022 [46]
Sparse OCT	No	No	Compressed sensing (CS) in spectral domain optical coherence tomography	To provide an elderly- and cost-friendly self-monitoring OCT	Maloca et al. 2018 [54]
SELFF OCT	No	No	off-axis, full-field, time-domain OCT	Reduce device complexity and cost	Burchard et al. 2022 [51].
SmartOCT	No	No	line-field OCT (LF-OCT)	Real time OCT imaging integrated to smartphone	Malone JD, Hussain et al. 2023 [55]

### 2.2. Home OCT of Fluid Monitoring Changes

#### 2.2.1. Device Variations for Fluid Monitoring

For both AMD and DME, early treatment with anti-vascular endothelial growth factor (anti-VEGF) has been shown to effectively reverse the development of neovascularization and macular edema [33,56,57]. Treatment typically involves an initial series of monthly injections, requiring an in-office visit, with retreatment based on visual stability and OCT findings. OCT is an important monitoring method for both early identification and customized treatment [37,58].

Although anti-VEGF therapy has been a cornerstone in treating neovascular AMD and DR, the progressive nature of these diseases places a significant burden on both patients and the healthcare system. The need for frequent office visits, multiple treatments, and additional testing contribute to this strain, with high discontinuation rates of anti-VEGF therapy—up to 57% in the first year [59].

The advancement of OCT technologies like the Foresee Home AMD, NVHO/Scanly, and SELFF OCT not only allows for early detection but also monitors fluid or biomarker changes to modify treatment accordingly [60]. ForeseeHome primarily supports the early identification of progression from dry to neovascular AMD, while device like Scanly enables patients to monitor retinal fluid changes in real time at home between clinic visits. In a 2022 study by Liu et al., the NVHO device, supported by the NOA AI system, showed strong agreement with expert analysis in detecting intraretinal fluid, comparable to in-office OCT [14]. SELFF-OCT represents another emerging tool, designed for affordable self-imaging, particularly useful in underserved or remote settings. While these devices are not yet designed to fully replace in-clinic imaging, they provide a reliable method for interim monitoring, and their use has been associated with fewer required treatments and extended treatment intervals [61].

#### 2.2.2. Portable OCT Advancement

A major obstacle to OCT systems’ portability has been their size. In the past years, the OCT has transitioned from bulky, stationary setups requiring significant space and desktop computers, making them unsuitable for screening in remote areas, to a more portable system housed in a cart, which currently represents the smallest design that includes all necessary components. Currently, several key approaches are being explored to make OCT systems more compact, focusing on optimizing both imaging quality and device size. These include handheld probes, home/self-OCT, and photonic integrated circuit (PIC)-based OCT, which can integrate complex components into a more compact design. Additionally, there is a portable OCT system based on a single-board computer (SBC-OCT) that was manufactured using a 3D printing system with a temperature-insensitive (TI) spectrometer. All hardware components, excluding optical parts, were made with a 3D printer [62,63].

Another advancement in data processing and real-time imaging technology is the development of portable boom-type ophthalmic UHR-OCT systems, allowing OCT images to be taken while lying down. The newer device, the ACT100, along with vertical-cavity Surface-emitting Lasers (VCSELs), enables faster imaging with an impressive acquisition speed of 350,000 scans per second. Furthermore, its integration with OCT instruments reduces patient chair time and visit duration, provides more efficient use of clinical space, and enhances the system’s performance [11,64,65,66].

As discussed above, another groundbreaking advancement in debulking OCT devices is the integration of smartphones with OCT, such as smartOCT. Another similar device is the Ocular CellScope, a retinal camera made up of a smartphone, a special housing with the necessary optics, and a phone holder to align the camera properly [55,67].

#### 2.2.3. Impact on Treatment Paradigms and Clinical Implications

Two common treatments for retinal conditions are Pro Re Nata (PRN), or “as needed”, where patients are monitored at fixed intervals with OCT and treated only when retinal fluid is detected, and treat-and-extend (T&E), where treatment intervals are extended when fluid resolves and shortened if it recurs or worsens [68,69].

A study by Heier et al. found that retina specialists often recommended delaying treatment when using home OCT data compared to relying on standard in-clinic OCT [70]. This suggests that home OCT data could provide a more real-time view of disease progression, leading clinicians to appropriately postpone treatment in certain cases. Home OCT tends to lean more towards the PRN model because it allows patients to monitor their condition more frequently and share the results with their healthcare providers when there are indications of disease progression. Home OCT could offer a clearer picture of the patient’s retinal condition in real-time, potentially allowing for more conservative management. This is supported by Holekamp et al., who highlighted that home OCT can potentially lengthen treatment intervals for AMD, showing significantly reduced treatment burden while maintaining stable visual acuity [61]. Additionally, retinal fluid fluctuations may not always be visible on the day of a scheduled in-clinic OCT scan. The pre-scheduled visits designed to detect new cases of CNV lesions were not very effective, underscoring the limitations of relying exclusively on in-clinic monitoring for tracking disease progression [24]. While home OCT provides enhanced, real-time monitoring of fluids, pairing it with regular in-office appointments for diagnosing other eye issues and guiding treatment plans remains crucial.

#### 2.2.4. Regulatory, Compliance, and Responsibility

As OCT technology moves from the clinical setting into patients’ homes, regulatory oversight and responsible use become increasingly important. While portable and home-based OCT devices promise to expand access and improve early detection, its integration with the expert operators introduces challenges in regulation, data security, and usability. As we embrace these advancements, we must navigate the complex regulatory compliance and responsibility to ensure safe and effective use, including medicolegal aspects.

The FDA oversees the approval of medical devices in the United States. Home OCT systems must undergo the FDA’s regulatory framework, depending on their intended use and the risk they pose. For example, the FDA recently granted de novo marketing authorization to Notal Vision’s artificial intelligence (AI)-powered Scanly Home OCT device [60]. Several other home-based OCT devices are still under clinical investigation.

Advancements in AI and cloud storage simplify data access for patients and doctors. Home OCT devices allow real-time imaging to detect retinal fluid and disease biomarkers. When fluid levels change or early signs appear, the device sends alerts through a digital system. In the NVHO system, for example, retina specialists set a fluid threshold for each patient. If the fluid exceeds that level, the system alerts a monitoring center, which notifies the specialist [50]. The specialist also reviews the patient’s home OCT images monthly, allowing for early detection and timely adjustments to treatment.

A key part of an ophthalmologist’s work includes the use of diagnostic tools and the evaluation of imaging. This necessitates extensive data storage, and with advances in technology, more of this data storage is moving onto the internet. Cloud-based systems have become responsible for ensuring data encryption and protection from unauthorized access [71]. While healthcare providers may access the data for diagnosis and treatment planning, the patient generally retains ownership of the data. However, this shift to cloud-based health data raises important concerns about data ownership and patient confidentiality. This is where informed consent and shared decision-making with patients are needed, understanding how their data will be collected, stored, and shared with healthcare providers. Patients may share their OCT images with healthcare providers through secure platforms that adhere to the Health Insurance Portability and Accountability Act (HIPAA), ensuring that only authorized physicians can access the data. For instance, with the Scanly device, physicians can review data, set specific criteria such as volume thresholds, and receive notifications via a HIPAA-compliant web portal [60].

Home OCT is designed for patients to use independently, minimizing the need for an operator. Patients receive instructions either through video or direct guidance on how to properly operate the device. The process requires the patient to position their head correctly and look through the device’s eyepiece with minimal movement to capture high-quality images [51,54,72]. The study by Yu et al. in 2021 found that the ability to use home OCT devices like ForeseeHome is promising, with a high success rate in patients who can manage the setup and imaging process. However, factors such as age, visual function, and specific conditions like geographic atrophy can impact the success rate [51,72,73]. While many patients can successfully use the device, considerations need to be made for those with age-related challenges or severe visual impairments.

In a traditional setting, trained technicians manage OCT imaging, and doctors manage interpretation. While most providers are open to engaging with telemedicine applications, some still express significant hesitations regarding current telemedicine practice modalities [74]. Compounding on this issue, home-based OCT could potentially produce images that are unclear or incorrect, which could result in misdiagnosis or delayed treatments. Although telehealth skills can be taught and assessed during medical education [75] to minimize the risk of mistakes by providers, patients should continue to combine in-person and remote care for optimal treatment, ensuring open communication with their physicians.

#### 2.2.5. Medicolegal Aspect

Although technology has been created to simplify workflow and already has a significant impact on healthcare, it carries several risks, such as “hackers” potentially gaining access to the host system and the insecure or incomplete deletion of health data [76]. The primary medico-legal concern surrounding mobile screening and mobile monitoring is that electronic medical records systems are largely unregulated in the US [77]. Security is a primary concern in cloud-based computing, making it essential to obtain informed patient consent regarding data governance and regulation. This should include a clear explanation of how the device functions, potential risks, and how medical records will be stored, including the duration of storage and who will have access. Data-sharing protocols should be detailed, particularly since the technology uses a cloud-based system. A data breach may significantly decrease client trust, which could later affect compliance.

#### 2.2.6. Pitfalls and Challenges

a. Patient and doctor experience and accessibility.

Current anti-VEGF treatment protocols place a significant burden on patients. The ongoing development of home OCT aims to reduce the necessity for frequent clinic visits for follow-up appointments, ultimately saving time, effort, and costs [70]. This convenience is particularly beneficial for elderly patients or those with limited mobility or transportation limitations. Another key factor contributing to patient satisfaction is the ease with which they can monitor their retinal condition from home. Conventional OCT requires steady alignment and limited movement, posing challenges for elderly patients or those with difficulty maintaining posture. Given that mobile OCT aims to simplify screening and monitoring, it should also be applicable for the elderly, as they are the most commonly affected age group by AMD and DR.

From the physician’s perspective, telemedicine and remote monitoring bring both opportunities and challenges. While many providers are optimistic about the rise of telemedicine, some still express doubt towards the development [74]. Although there are no specific studies measuring physician satisfaction with this new technology, improved patient outcomes and the ability to monitor conditions more closely to prevent vision loss may indirectly serve as indicators of physician satisfaction [24,48,61,72]. However, the need for more training in data interpretation and remote monitoring remains a key barrier to widespread adoption.

Additionally, the quality of self-acquired images is a crucial factor influencing both physician confidence and clinical decision-making; poor-quality scans can lead to misinterpretation, complicate diagnosis, and hinder effective patient care. Therefore, adapting and improving OCT technology while ensuring the quality of images and maintaining diagnostic accuracy is essential.

Patient compliance plays a central role in the success of home OCT. A study by Holekamp et al. demonstrated strong patient adherence to self-imaging protocols over a six-month period, with consistent weekly scan frequencies and no significant drop-off in usage [61]. This suggest that with user-friendly design, patients are highly capable of integrating home OCT into their routine care.

The potential of remote imaging technologies reaches far beyond just helping individual patients—it has real power to impact public health on a much larger scale. For instance, remote retinal imaging has led to increased diabetic retinopathy screenings [78]. The use of home OCT and AI-based systems offers more opportunities to further decentralize screening, making care more accessible. However, global implementation remains uneven, especially in areas with limited healthcare infrastructure. In Indonesia, for instance, less than 25% of individuals with Vision-threatening Diabetic Retinopathy (VTDR) have received optimal treatment [79]. Due to the geographic constraints of Indonesia, it becomes important to include general practitioners in ocular screening, such as DR. However, beyond the deficiency in appropriate screening facilities and the knowledge and skills required, low compliance and socioeconomic issues are still problems that contribute to the low screening numbers [80]. This is where the implementation of home-based OCT is being questioned.

Although portable OCT could greatly benefit developing countries with limited access to specialists and challenging geography, implementing home OCT is not without challenges. Successful application will depend on the devices being affordable, easy to use, and supported by adequate training and healthcare infrastructure. Cost-effectiveness and ease of use must be carefully assessed to ensure the technology benefits all populations.

b. Reimbursement issues

While remote retinal imaging has become more widely used, reimbursement remains inconsistent, especially for specific imaging codes, and the gap in payments for these services is growing [81,82]. While some devices, such as the ForeseeHome device (Notal Vision), are covered by Medicare and most private insurers, the reimbursement system for other home-based OCT devices is still unclear [45]. Although the purpose of home and portable OCT devices is to reduce treatment costs, several factors need to be considered, such as accessibility and availability, that potentially increase costs.

c. Challenges in device resolution, image quality, and data accuracy

When it comes to miniaturization, making devices more compact, and enhancing their functions, there are trade-offs in image quality that must be considered. For example, while sparse OCT offers faster imaging, its quality is lower compared to high-resolution SDOCT, especially in terms of smoothness and noise reduction [54]. SELFF OCT also showed higher background noise, but it still defined retinal layers clearly in most cases, despite challenges like reflected and scattered light [51]. On the other hand, the NVHO 2.5 device produced high-quality images, with 99% of them deemed usable by doctors, which is nearly identical to commercial OCT. This device was highly accurate in detecting fluid in the eye, such as SRF and IRF, with very high agreement rates (98% for any fluid, 93% for SRF, and 91% for IRF). Moreover, its diagnostic accuracy remained consistent across different levels of visual acuity, making it a reliable tool for home use [72].

## 3. Intraoperative OCT in Vitreoretinal Surgery

### 3.1. Instrumentation and Background

Perhaps the most applied use of OCT outside the clinic, particularly over the past decade, has been in the operating room with iOCT. Traditional microscope viewing systems in ophthalmic surgery restrict a surgeon’s view to an *en face* perspective, relying on surgeon depth-of-field approximation and limiting any cross-sectional views. This long-standing shortcoming, combined with rapidly improving OCT technology in the early 2000s, created opportunities for novel intraoperative imaging solutions. The earliest reports of iOCT involved modifications of existing tabletop OCT systems for use in supine patients [83,84]. Other early reports of intraoperative OCT use involved modified tabletop systems for examination under anesthesia [85]. While modified tabletop systems demonstrated the potential for integrating OCT into the operating room, their fixed configuration and spatial limitations restricted access to the sterile surgical field, lacking practicality and disrupting surgical workflow [86].

A significant leap in the progression of iOCT came with the introduction of portable OCT scan heads in the late 2000s. The two most commonly described handheld systems in literature are the Bioptigen SDOIS/Envisu (Bioptigen, Research Triangle Park, Morrisville, NC, USA) and the Optovue IVue (Optovue, Fremont, CA, USA). Both handheld systems utilize SD-OCT. The handheld systems solved the problem of portability that was lacking in traditional tabletop systems and were also modified for external and microscope mounting to reduce motion artifacts from freehand handheld capture [86]. The landmark PIONEER study conducted by Ehlers et al. investigated the safety and utility of mounted handheld iOCT devices in anterior and posterior segment surgeries [87]. Intraoperative imaging was obtained in 98% of the 531 total cases over the first 24 months of the study. The study found that iOCT altered surgical decision-making in approximately 48% of lamellar keratoplasty cases and 43% of membrane peeling procedures—with a median capture time of 4.9 min per scan session [87]. This study demonstrated the feasibility and utility of iOCT in ophthalmic surgery. However, shortcomings, including the need to pause surgery for imaging, the need for ancillary technician support, and the lack of real-time feedback, necessitated the integration of the OCT with the operating microscope [87].

To overcome limitations of handheld and microscope-mounted OCT systems, microscope-integrated intraoperative OCT (iOCT) devices were developed. Unlike earlier systems that operate independently from the surgical microscope, integrated iOCT shares the same optical axis, minimizing workflow disruption [88,89]. Three systems are currently available: the Zeiss RESCAN 700 [90], Leica EnFocus Ultra-HD [91], and Haag-Streit iOCT [92]. The RESCAN 700, FDA-approved in 2014, features SD-OCT technology with a 27,000 A-scan/second rate and 5.5 µm axial resolution, giving advantage of the total structural and ergonomic integration of OCT into the microscope, which avoids disturbing the outline of the microscope compared to previous integrated iOCT attempts [86]. The EnFocus, approved in 2015, offers >36,000 A-scans/second and 4.0 µm resolution, with added touchscreen and heads-up display capabilities.

The DISCOVER study, also conducted by Ehlers et al., sought to investigate the feasibility and impact of these microscope-integrated iOCT systems, including the RESCAN 700 and EnFocus, on surgical decision-making. Of the 837 eyes enrolled, 820 were successfully imaged with iOCT. Microscope-integrated iOCT altered surgical decision-making in 43.4% of anterior segment cases and 29.2% of posterior segment cases. Along with the reported changes in surgical decision-making, the results of the DISCOVER study also revealed several areas of potential improvement in iOCT implementation. For one, metallic surgical instrumentation was observed to cause significant shadowing, leading to suboptimal instrument visualization during real-time imaging [88], highlighting the need for OCT-compatible instrumentation. A subsequent study by Ehlers et al. described the potential of semitransparent surgical instrumentation (i.e., picks, vitreoretinal forceps) during real-time OCT imaging with the RESCAN 700 and EnFocus system [93]. This study noted improved shadowing in the semitransparent instrumentation but illustrated the need for ongoing refinement of visualization software and specific tools, especially those with complex geometry like vitreoretinal forceps [94]. Other notable areas of potential improvement gleaned from the DISCOVER study included optimization of surgeon heads-up-display, automated OCT aiming, and improved software for imaging analysis [88,95]. Implementation of SS-OCT in iOCT, while currently not in widespread use, also offers promise for faster acquisition speeds, live volumetric visualization, automated OCT tracking, and widefield OCT imaging [96,97,98].

While experimental integration of OCT into needle-based probes and into surgical instrumentation itself has been described [99,100,101], most clinical studies investigating the benefit of iOCT in ophthalmic surgery have utilized microscope-integrated OCT (Table 2). There is a growing body of evidence emphasizing the potential role for intraoperative OCT in image-guided surgery and the potential effects on patient outcomes and surgical decision-making. The following sections cover a variety of anterior and posterior segment conditions and the impact iOCT has on surgical decision-making and outcomes.

### 3.2. Anterior Segment OCT Use in the OR

Anterior Segment Optical Coherence Tomography (AS-OCT) is an advanced imaging modality that provides high-resolution cross-sectional images of the anterior segment of the eye. The application of intraoperative OCT (iOCT) in the operating room, whether handheld or microscope-integrated (miOCT) has significantly enhanced intraoperative decision-making, allowing for precise visualization of ocular structures, improved surgical outcomes, and better patient care. The benefits of AS-OCT use in the OR are shown particularly in corneal and ocular surface diseases, anterior segment imaging, anterior chamber and angle surgery, and refractive surgery.

**Figure 6:**
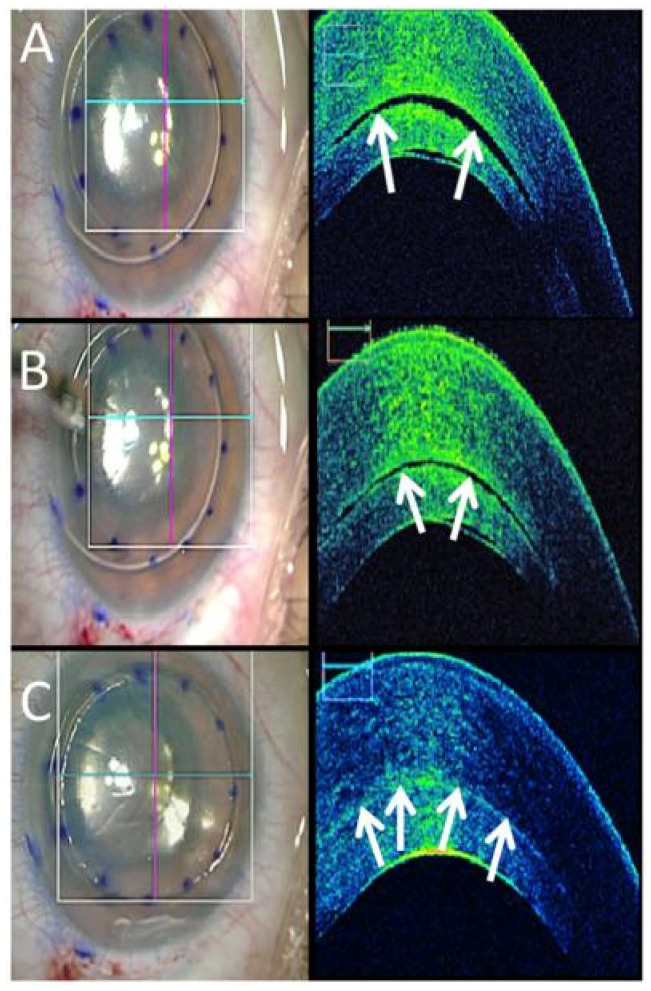
Time lapse of iOCT DSAEK. (**A**) En face image after air-bubble infusion (**left**), with B-scan (**right**) showing interface fluid (arrows). (**B**,**C**) After corneal massage, en face appearance remains stable (**left**), while B-scans (**right**) demonstrate reduction in interface fluid (arrows). Image by Ehlers et al. from Determination of feasibility and utility of microscope-integrated optical coherence tomography during ophthalmic surgery: the DISCOVER Study RESCAN Results [130].

iOCT and miOCT play a crucial role in managing corneal diseases and ocular surface disorders by providing detailed structural imaging at the time of surgery. During corneal transplantation procedures such as penetrating keratoplasty (PKP) [102] and Descemet’s stripping automated endothelial keratoplasty (DSAEK) (Figure 6) [103], iOCT allows for real-time assessment of graft-host interface alignment, ensuring better graft positioning and adherence [104,105]. It also aids in evaluating corneal thickness in keratoconus patients undergoing Corneal Cross-linking (CXL) [105,106] and assists in monitoring epithelial remodeling after phototherapeutic keratectomy (PTK) [107]. In ocular surface diseases, AS-OCT/iOCT helps in assessing conjunctival scarring [108], limbal stem cell deficiency, and the effectiveness of amniotic membrane transplantation. It is also instrumental in dry eye disease by measuring tear film thickness and assessing changes in meibomian gland dysfunction, allowing for more tailored treatment approaches [109,110,111].

iOCT also provides visualization of the deeper anterior segment structures, allowing for enhanced intraoperative precision. The imaging modality is particularly beneficial in cataract surgery [12], where it helps in evaluating anterior chamber depth, lens thickness, and capsular integrity before and during phacoemulsification. In femtosecond laser-assisted cataract surgery (FLACS) [112], iOCT guides laser positioning for capsulotomy, corneal incisions, and lens fragmentation [113], improving surgical accuracy and patient outcomes. Moreover, iOCT aids in detecting and managing intraoperative complications such as posterior capsule rupture, zonular dehiscence, and corneal wound integrity, allowing surgeons to make real-time adjustments [114]. SS-OCT is also valuable in assessing intraocular lens (IOL) positioning post-implantation, reducing the risk of refractive surprises and visual distortions [115].

Anterior chamber and angle surgery, particularly glaucoma procedures, also benefit from iOCT. In minimally invasive glaucoma surgeries (MIGS) such as trabecular microbypass stents and canaloplasty, AS-OCT provides high-resolution imaging of the iridocorneal angle, Schlemm’s canal, and trabecular meshwork, ensuring accurate device placement and improved surgical outcomes [116]. For traditional glaucoma surgeries like trabeculectomy and glaucoma drainage device implantation, AS-OCT helps in assessing bleb morphology [117,118], scleral flap positioning [119], and visualizing the aqueous outflow tract. This allows for early detection of complications such as fibrosis [120], bleb leaks [120], and resulting hypotony [121], facilitating timely intervention and optimizing long-term intraocular pressure (IOP) control.

AS-OCT also aids in managing anterior segment trauma cases [122], allowing for precise evaluation of angle recession, iridodialysis, and cyclodialysis clefts. By providing real-time structural assessment, it guides the decision-making process regarding surgical repair and long-term management.

In refractive surgery, AS-OCT enhances preoperative assessment, intraoperative precision, and postoperative monitoring. It is particularly valuable in laser vision correction procedures such as LASIK, PRK, and SMILE, where it provides detailed corneal thickness mapping, epithelial profiling, and flap interface evaluation. This helps in customizing treatment plans based on individual corneal biomechanics, reducing the risk of postoperative complications such as ectasia and irregular astigmatism [123]. During phakic IOL implantation, AS-OCT assists in measuring anterior chamber depth and angle anatomy, ensuring proper lens sizing and positioning [124]. In refractive lens exchange procedures, it aids in selecting the appropriate IOL power by providing precise biometric data [123]. Intraoperative OCT has proven valuable for challenging lenticule extractions in SMILE, by offering real-time imaging of the lenticule and clarifying its positioning relative to the anterior stromal cap and underlying stromal bed. Utilizing iOCT has led to favorable anatomical and visual results [125].

Postoperatively, AS-OCT is useful in detecting early signs of complications such as flap displacement [126], epithelial ingrowth [127], and corneal haze [128]. For CXL procedures for keratoconus, AS-OCT enables post-operative assessment of corneal stromal demarcation lines at two weeks, ensuring adequate riboflavin penetration and UV exposure [129]. Its non-contact nature makes it ideal for serial monitoring without causing patient discomfort or disrupting the corneal surface/tear film, leading to better long-term refractive stability and visual outcomes.

### 3.3. Posterior Segment OCT Use in the OR

Epiretinal membranes (ERMs) are fibrocellular proliferations that form on the inner surface of the retina, often occurring idiopathically or following posterior vitreous detachment, retinal laser procedures, surgery, or uveitis [131]. OCT is instrumental in diagnosing ERMs, typically revealing a hyperreflective membrane on the retinal surface, accompanied by retinal thickening, surface wrinkling, and sometimes cystoid spaces, which altogether help to assess the severity and guide treatment decisions [131]. Visually significant ERMs are among the most common indications for vitrectomy and are treated with membrane peeling, which relieves vitreoretinal traction. ERMs are also among the best-characterized vitreoretinal diseases on OCT, which contributes to the high frequency at which iOCT is indicated in membrane peeling procedures in reported clinical studies [87]. Intraoperative OCT has demonstrated utility in nearly every step of membrane peeling surgery from start to finish—from improved engagement of the posterior hyaloid, to increased tissue reflectivity with the use of indocyanine green and triamcinolone [132,133]. Furthermore, both the previously mentioned PIONEER and DISCOVER studies revealed a discordance between surgeon identification and iOCT identification of residual membranes in 13–22% of peel cases, and iOCT was able to identify the extent of membranes that were unseen by the surgeon. In contrast, in 15–40% of cases where the surgeon suspected a residual membrane, intraoperative OCT confirmed complete removal, avoiding unnecessary surgical intervention [87,95].

In addition to optimizing surgical efficiency, iOCT may offer an alternative to dye and contrast based chromovitrectomy. Indocyanine green remains one of the most commonly used agents in membrane peeling procedures, despite its reported retinal toxicity [134] and potential damage to the optic nerve [135], RPE, and retinal nerve fiber layer [136]. With no reported differences in visual outcomes, dye-free peeling has been achieved in around 40–60% of membrane peeling cases in reported clinical studies by Falkner-Radler et al. and Leisser et al. [137,138]. Overall, iOCT has demonstrated utility in impacting surgical decision-making in membrane peel surgery.

**Figure 7:**
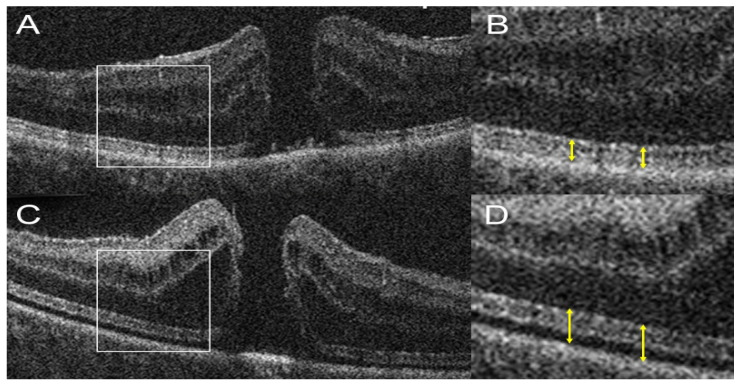
Intraoperative OCT images of a macular hole (MH) using the Bioptigen SDOIS system. White boxes in (**A**,**C**) denote regions magnified in (**B**,**D**) respectively. (**A**,**B**) Before ILM peeling, outer retinal layers including the ellipsoid zone (EZ) are intact, with normal EZ–RPE distance (yellow double arrow). (**C**,**D**) After ILM peeling, MH depth increases slightly, and EZ–RPE height expands, suggesting shallow retinal detachment. Image from Ehlers et al. Predictive Model for Macular Hole Closure Speed: Insights From Intraoperative Optical Coherence Tomography [143].

Macular holes (MH) are full-thickness defects in the neurosensory retina at the macula, leading to central vision loss. Pathogenesis often involves vitreoretinal interface abnormalities, where anomalous posterior vitreous detachment exerts tractional forces, culminating in retinal tissue disruption [139]. OCT has been instrumental in our understanding and characterization of the formation of MHs, revealing a predictable sequence of events from initial posterior vitreomacular traction to full-thickness macular hole formation [140]. Surgical intervention, primarily pars plana vitrectomy with internal limiting membrane (ILM) peeling and gas tamponade, achieves anatomical closure rates exceeding 90% [141]. As with epiretinal membrane peeling, iOCT has offered novel insights into tissue alterations and tractional dynamics during ILM peeling in macular hole surgery [142]. Using iOCT, Ehlers et al. identified several predictors for early macular hole closure, most notably pre-incision minimal width [143]. Intraoperative changes in macular hole volume and minimal width were also identified as predictors of early macular hole closure, the subtle increase in MH depth and expansion of the EZ–RPE distance following ILM peeling may indicate early retinal mobilization and localized detachment (Figure 7) [143]. Another group reported a novel intraoperative sign on iOCT, named the “hole-door sign”, which was associated with a higher likelihood of achieving postoperative Type-1 macular hole closure, indicating successful anatomical repair [144]. This sign is characterized by vertical pillars of tissue at the edges of the macular hole, projecting into the vitreous cavity after ILM peeling. Intraoperative OCT-confirmed MH closure may also offer a shorter face-down positioning time post-surgery [145]. These studies serve as examples of how iOCT findings can serve as predictive markers for surgical success, enhancing decision-making during MH surgery and guiding counseling for prognosis after surgery.

The risk of vitreous macular traction (VMT) progressing to a FTMH carries significant implications for prognosis [146]. Traditional surgical treatment for VMT involves vitrectomy, which may be combined with membrane peeling or other techniques [147]. The success of such surgeries relies on precise identification and treatment of the areas of traction. iOCT provides real-time imaging in high-resolution, cross-sectional images of the macula and retina during surgery. iOCT allows surgeons to directly observe the extent of traction, monitor surgical progress, and guide intraoperative decisions with greater accuracy [148].

Rhegmatogenous retinal detachment (RRD) is characterized by the separation of the neurosensory retina from the underlying RPE due to a full-thickness retinal break. Primary surgical repair success rates for RRD vary depending on the chosen technique and patient-specific factors, but comparative studies have reported primary repair success rates from 84% to 94% [149]. The primary goal in the surgical repair of RRD—either through scleral buckling, PPV, or pneumatic retinopexy—is to reattach the retina by closing all retinal breaks and alleviating vitreoretinal traction. According to a post hoc analysis of the DISCOVER study by Abraham et al., iOCT provided valuable feedback in 36% of total RD cases, particularly in complex RD cases (50%), and altered decision making in 12% of cases [148]. iOCT has also been used to characterize and visualize microstructural changes during RRD repair surgery in several studies [150,151,152]. A case series conducted by Ehlers et al. describes a series of novel iOCT findings during combined vitrectomy/scleral buckle for nine eyes [152]. Specific reported findings include subfoveal hyporeflectivity followed by subretinal hyporeflectivity and foveal thinning with MH formation, and finally, overt full-thickness MH [152]. Other studies identified residual subretinal fluid after PFO injection using iOCT but noted no correlation between the fluid and functional or anatomical outcomes [150,151]. The proposed benefits of iOCT in retinal detachment repair surgery are not as elucidated as in membrane peeling surgeries but still seem to augment surgical decision-making in some capacity [148,153].

**Figure 8:**
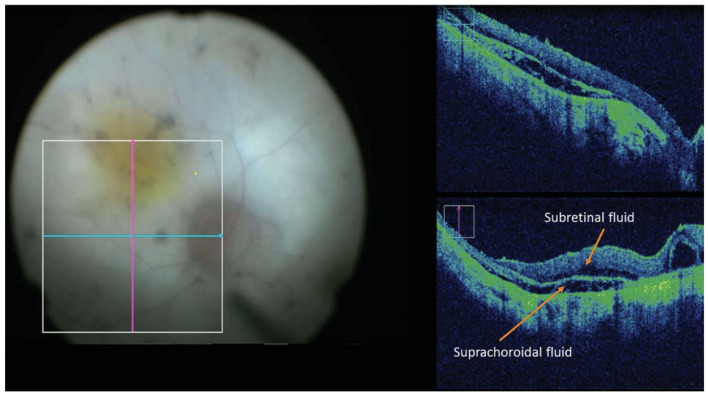
Intraoperative microscope-integrated OCT (RESCAN 700) image of raised subretinal bleb not readily detectable on microscope fundus view. Image by Gregori et al. from Intraoperative Use of Microscope-Integrated Optical Coherence Tomography for Subretinal Gene Therapy Delivery [154].

Intraoperative OCT also offers significant potential in enhancing the precision and safety of subretinal therapeutic procedures. Of particular interest is the subretinal delivery of gene therapy for inherited retinal conditions such as Leber’s Congenital Amaurosis and Retinitis Pigmentosa. Due to the relatively impermeable nature of the ILM, the potential for off-target transduction, and to facilitate direct delivery to the RPE and photoreceptors, subretinal injection is the preferred delivery method for gene therapy. By providing real-time, high-resolution cross-sectional imagery of delicate subretinal anatomy, iOCT allows surgeons to visualize and monitor the creation and expansion of the subretinal bleb—the localized retinal detachment where therapeutic agents are delivered (Figure 8) [154,155,156,157]. Insights from the previously discussed PIONEER and DISCOVER studies have also demonstrated iOCT-facilitated localization of subretinal injection of tPA in cases of subretinal hemorrhage [158], iOCT-assisted chorioretinal biopsy [93], and iOCT-facilitated identification and removal of retained perfluorocarbon liquid droplets in a case of persistent subretinal PFO [159]. A recent case series by Valikodath et al. also utilized iOCT to obtain volumetric measurements of injected subretinal tPA and found that volumetric iOCT measurements differed from volumetric measurements from the actual syringe [160]. The authors suggest that this discrepancy could be a result of leakage from the retinotomy into the vitreous or delivery outside the eye from human error. These studies point to the conclusion that iOCT can facilitate objective and more accurately reproducible measurements for subretinal therapeutic delivery.

**Figure 9:**
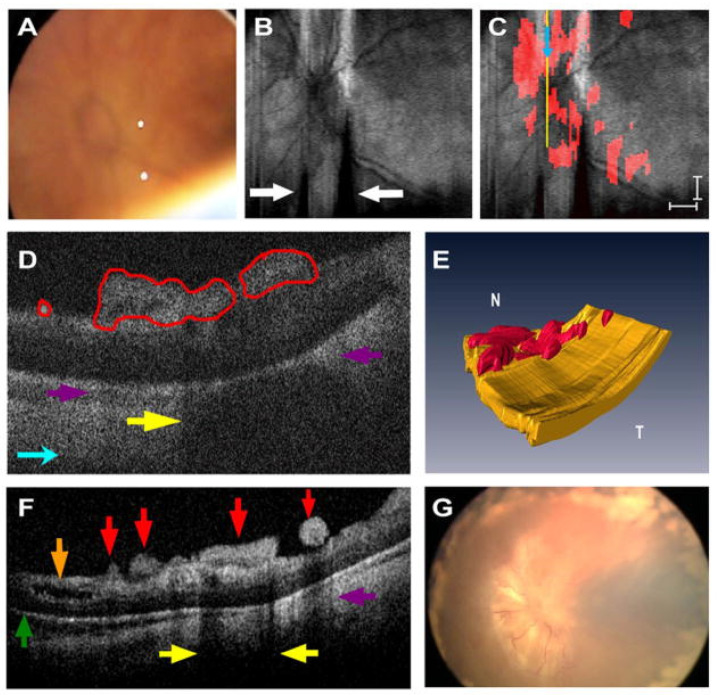
Imaging of the left eye of a former 650-g, 23-week postmenstrual age (PMA), Caucasian twin male who received confluent laser treatment to both eyes at 32 weeks PMA. Handheld SD OCT imaging was performed without sedation in both eyes within 24 h of the clinical examination: (**A**) Indirect video image shows the optic nerve and posterior retina without visible preretinal tissue. (**B**) Summed voxel projection reveals the optic nerve and posterior pole; white arrows indicate motion artifacts. (**C**) Same projection as in (**B**), with red-highlighted preretinal tissue surrounding and overlying the optic nerve, as seen in (**D**); light blue arrows indicate orientation. Scale bars = 1000 μm. (**D**) Cropped SD-OCT cross-section (aligned with the yellow line in (**C**)) shows preretinal structures outlined in red. Purple arrows indicate shadowing from these structures, and yellow arrows show focal shadowing. (**E**) 3D SD-OCT rendering reveals preretinal membranes at the posterior pole (N = nasal; T = temporal). (**F**) Images revealed clinically undetected preretinal structures and retinoschisis in both eyes and a very shallow retinal detachment in the left eye. Cross-sectional OCT displays multiple subclinical findings: the green arrow indicates retinal detachment, the brown arrow shows retinoschisis, the yellow arrow highlight focal shadowing and the red arrows highlight preretinal tissue. (**G**) Fundus photo (Retcam) taken one month later confirms tractional retinal detachment. Image by Chavala et al. from Insights into Advanced Retinopathy of Prematurity Using Handheld Spectral Domain Optical Coherence Tomography Imaging [164].

In pediatric patients, retinal imaging is often challenging and requires particular attention to developmental considerations that are not present in adult populations. The most practical challenge of performing OCT in pediatric patients is a lack of cooperation and poor fixation. Traditional tabletop OCT systems require upright positioning onto a chinrest, which precludes accurate capture in resistant patients as well as infants who are unable to support themselves. Pediatric patients also possess varied and more dynamic ocular proportions compared to adults, including shorter axial lengths, shorter corneal diameter, and higher astigmatism, which can complicate imaging [161,162]. Varied foveal anatomy in correlation with gestational age must also be taken into consideration when interpreting OCT findings in preterm infants [163]. The advent of handheld OCT and later microscope-integrated SD-OCT and OCT-A address these shortcomings and have provided greater insight into the pathophysiology and assessment of a variety of pediatric retinal conditions. In retinopathy of prematurity (ROP), handheld OCT can reveal pathological findings not readily identifiable (Figure 9) on clinical examination such as retinoschisis, retinal detachment, and preretinal alteration [164]. This may offer some benefit as an adjunct to indirect ophthalmoscopy in ROP screening in at-risk infants. Intraoperative OCT also enables monitoring of neovascularization in aggressive posterior ROP and can assist in directing targeted photoablation in the posterior retina [165]. In retinoblastoma (RB), Handheld OCT can be used to monitor treatment response pre-and-post chemotherapy [166]. One report by Malik et al. describes a case of treatment-resistant retinoblastoma and the utility of handheld OCT in the longitudinal evaluation and submillimeter characterization of tumor recurrence [167]. Intraoperative handheld and microscope-integrated OCT has also conferred utility in cases of pediatric trauma [155] and tractional pathology [168].

An emerging application of iOCT, intraoperative OCT angiography (iOCTA), has also demonstrated particular utility in pediatric retinal disorders. In a report by Chen et al., two pediatric patients with idiopathic vitreous hemorrhage and familial exudative vitreoretinopathy underwent examination under anesthesia with microscope-integrated iOCTA. This report demonstrated increased visualization and greater detail of vasculature on iOCTA compared with conventional fluorescein angiography [169]. Additionally, a cross-sectional study of infants with persistent fetal vasculature who underwent examination under anesthesia using iOCTA revealed characteristic but subtle vascular and flow abnormalities that were less visible with fluorescein angiography [170]. It is evident that iOCT and iOCTA not only confer benefit in their ability to examine non-cooperative patients but also in the improved characterization and monitoring of pediatric retinal vascular disorders, oncologic, and tractional disorders.

**Table 2:** Summary of intraoperative OCT uses in selected ocular disorders.

Disease	Procedure/Treatment	iOCT Type	Benefits	References
Keratoconus	Corneal Collagen Cross Linking	Handheld	- Intraoperative pachymetry	Rechichi et al., 2014 [106]
Primary Open Angle Glaucoma	MIGS	Microscope-Integrated	- Improved visualization of iridocorneal angle, Schlemm’s canal, and trabecular meshwork.	Kan et al., 2022 [116]
Epiretinal Membrane	Epiretinal Membrane Peeling	Microscope-Integrated	- Enhanced visualization of chromovitrectomy dyes - Identification of residual membranes - Confirmation of membrane removal	Ehlers et al., 2011 Ehlers et al., 2014 Falkner-Radler et al., 2015 Leisser et al., 2016 [132,137,138]
Macular Hole	FTMH Repair	Microscope-Integrated	- Identification of novel prognostic markers during surgery	Ehlers et al., 2019 [142]
Rhegmatogenous Retinal Detachment	Pneumatic Retinopexy, Gas/Oil Tamponade	Microscope-Integrated	- Characterization of surgical tissue alterations	Lee et al., 2011 [150].
Inherited Retinal Diseases	Subretinal Injection	Microscope-Integrated	- Volumetric monitoring of subretinal injection - Confirmation of probe location	Gregori et al., 2019 [154]
Retinopathy of Prematurity	Screening, Photoablation	Handheld	- Novel morphological insights - Adjunct to routine screening - Photoablation targeting	Lee et al., 2011 [150]
Retinoblastoma	Chemotherapy	Handheld	- Monitoring of chemotherapy response and tumor recurrence	Malik et al., 2020 [167]

### 3.4. Challenges

The integration of OCT into the operating room requires consideration of financial and regulatory challenges. High acquisition and maintenance costs limit accessibility, particularly in resource-limited settings when cost-benefit analysis is yet to be determined [171]. In a post hoc economic analysis of the ADVISE trial, the cost-effectiveness of the iOCT protocol of DMEK surgery was evaluated and determined that there were no statistical differences in Quality Adjusted Life Year (QALY) and no superiority in incremental cost-effectiveness ratio, though there was a marginal difference in total costs per DMEK of €107 in favor of the iOCT protocol and a shorter mean net surgical time of 4.9 min [172]. This analysis of long-term cost savings in the > 6-month post-operative period.

The use of real-time imaging during surgery requires adherence to evolving guidelines and safety protocols, such as compliance with international standards IEC 60601-1 for medical electrical equipment safety and ISO 13485 for quality management. In some regions, obtaining regulatory approval for integrating OCT into surgical workflows may be a complex process, involving compliance with both medical device regulations and data protection policies. The lack of standardized protocols across institutions further complicates implementation. There is a need for universally accepted guidelines for intraoperative OCT use, including criteria for image acquisition, interpretation, and decision-making.

#### Does Intraoperative OCT Change Decision-Making?

While i-OCT provides highly detailed imaging and enhances surgical precision, the extent to which it changes intraoperative decision-making remains a subject of discussion. Studies suggest that while AS-OCT allows for real-time visualization of corneal, anterior chamber, and angle structures, its impact on surgical outcomes depends on the surgeon’s experience and the complexity of the procedure. Research has shown that intraoperative OCT may alter surgical decisions in the posterior segment in approximately 29.2% (PIONEER study in 2014) to 43% (DISCOVER study in 2018) of cases, and 26.2% in a smaller, pilot prospective case series conducted in 2022 at a tertiary institution in Lahore, Pakistan [173]. However, cost–benefit analyses from the societal perspective yield fewer clear results [172].

In corneal surgeries, intraoperative AS-OCT is beneficial in evaluating the graft-host interface in lamellar keratoplasty and guiding corneal incisions. However, some experts argue that experienced corneal surgeons can achieve similar outcomes without AS-OCT, using their clinical judgment and traditional microscopy.

AS-OCT facilitates precise device placement and angle assessment in anterior segment and glaucoma surgeries. While this enhances safety and efficiency, there is limited evidence to suggest that it significantly alters decision-making in most cases. Some surgeons may use intraoperative AS-OCT selectively rather than routinely, incorporating it only when standard visualization techniques are insufficient [12].

In refractive surgery, intraoperative AS-OCT assists in assessing corneal thickness and flap integrity. However, the extent to which it improves refractive outcomes compared to preoperative and postoperative imaging is still under investigation. Since many refractive surgeries already employ advanced imaging techniques preoperatively, the necessity of intraoperative OCT remains debatable [12].

In posterior segment surgery, iOCT has demonstrated a marked ability to impact decision-making, particularly in membrane peel surgeries for macular holes and epiretinal membranes. Likewise, its potential role in facilitating subretinal therapeutic delivery has also been demonstrated. While the impact of iOCT in procedures such as retinal detachment repair remains fully elucidated, iOCT has already established itself as a watershed advancement in the management of retinal disease.

## 4. Conclusions and Future Directions

Unprecedented understanding of the precipitating events in ocular pathology, new pharmaceuticals, and novel surgical techniques has driven demand for nontraditional OCT technologies capable of providing high-definition imaging and integrated AI-assisted analyses.

The introduction of OCT for use outside the clinic has been welcomed by patients and clinicians. Unlike the previous bulky and costly OCT devices, which had limited its availability to large medical facilities and restricted access for many patients, today’s OCT devices are transforming ocular disease management by placing imaging access in patients’ hands. With an increasing demand for simplicity and practicality, innovations such as home-based OCT now allow patients to monitor their eye health from the comfort of their own homes. Elderly patients and those with chronic conditions, particularly those with visual impairments, have already benefited from this innovative OCT technology. Home-based OCT patients have provided largely positive feedback and reportedly value the convenience. Intraoperative OCT has proven useful for surgeons and has changed surgical decisions in many cases, helping surgeons identify issues such as residual membranes or macular hole closure that may not be visible otherwise. In pediatric cases and other complex surgeries, iOCT and handheld OCT is improving surgical outcomes and pathology visualization and characterization. For home-based OCT and intraoperative OCT to reach a wider population, the high upfront costs and reimbursement challenges must be addressed. Further research is necessary to understand how best to address the problem of high cost so that this technology can transcend socioeconomic barriers and benefit wider, more diverse populations.

Another challenge involves standardizing protocols for OCT at home and when used during surgery. These protocols are important for ensuring the accuracy and consistency of results, especially AI-facilitated reports. Patients and healthcare providers should know who bears responsibility for misdiagnosis, including legal medical liability related to misinterpretation and mismanagement of results. Moving forward, the best OCT protocols will be developed through collaboration between patients, healthcare providers, healthcare institutions, manufacturers, and regulators.

While home OCT is proven to help monitor the progression of the disease, portable handheld OCT, such as the Act100 device and Bioptigen, may offer a more practical solution in remote areas. With professional oversight, the devices can be readily utilized in the primary care setting. This is especially beneficial for developing countries that have limited healthcare infrastructure. Further study with larger portable OCT sample sizes will be necessary to evaluate its screening effectiveness in diverse large populations and to assess its cost in real-world applications.

## References

[1] Huang D., Swanson E.A., Lin C.P., Schuman J.S., Stinson W.G., Chang W., Hee M.R., Flotte T., Gregory K., Puliafito C.A. (1991). Optical Coherence Tomography. Science.

[2] Fujimoto J.G., Brezinski M.E., Tearney G.J., Boppart S.A., Bouma B., Hee M.R., Southern J.F., Swanson E.A. (1995). Optical Biopsy and Imaging Using Optical Coherence Tomography. Nat. Med..

[3] Fujimoto J., Swanson E. (2016). The Development, Commercialization, and Impact of Optical Coherence Tomography. Investig. Ophthalmol. Vis. Sci..

[4] Gabriele M.L., Wollstein G., Ishikawa H., Kagemann L., Xu J., Folio L.S., Schuman J.S. (2011). Optical Coherence Tomography: History, Current Status, and Laboratory Work. Investig. Ophthalmol. Vis. Sci..

[5] Adhi M., Duker J.S. (2013). Optical Coherence Tomography—Current and Future Applications. Curr. Opin. Ophthalmol..

[6] Javed A., Khanna A., Palmer E., Wilde C., Zaman A., Orr G., Kumudhan D., Lakshmanan A., Panos G.D. (2023). Optical Coherence Tomography Angiography: A Review of the Current Literature. J. Int. Med. Res..

[7] Potsaid B., Baumann B., Huang D., Barry S., Cable A.E., Schuman J.S., Duker J.S., Fujimoto J.G. (2010). Ultrahigh Speed 1050nm Swept Source/Fourier Domain OCT Retinal and Anterior Segment Imaging at 100,000 to 400,000 Axial Scans per Second. Opt. Express.

[8] Pieroni C.G., Witkin A.J., Ko T.H., Fujimoto J.G., Chan A., Schuman J.S., Ishikawa H., Reichel E., Duker J.S. (2006). Ultrahigh Resolution Optical Coherence Tomography in Non-Exudative Age Related Macular Degeneration. Br. J. Ophthalmol..

[9] Yehoshua Z., Wang F., Rosenfeld P.J., Penha F.M., Feuer W.J., Gregori G. (2011). Natural History of Drusen Morphology in Age-Related Macular Degeneration Using Spectral Domain Optical Coherence Tomography. Ophthalmology.

[10] Spaide R.F. (2009). Age-Related Choroidal Atrophy. Am. J. Ophthalmol..

[11] Chopra R., Wagner S.K., Keane P.A. (2021). Optical Coherence Tomography in the 2020s—Outside the Eye Clinic. Eye.

[12] Titiyal J.S., Kaur M., Falera R. (2017). Intraoperative Optical Coherence Tomography in Anterior Segment Surgeries. Indian J. Ophthalmol..

[13] Mallipatna A., Vinekar A., Jayadev C., Dabir S., Sivakumar M., Krishnan N., Mehta P., Berendschot T., Yadav N.K. (2015). The Use of Handheld Spectral Domain Optical Coherence Tomography in Pediatric Ophthalmology Practice: Our Experience of 975 Infants and Children. Indian J. Ophthalmol..

[14] Liu Y., Holekamp N.M., Heier J.S. (2022). Prospective, Longitudinal Study: Daily Self-Imaging with Home OCT for Neovascular Age-Related Macular Degeneration. Ophthalmol. Retin..

[15] Dolar-Szczasny J., Drab A., Rejdak R. (2024). Home-Monitoring/Remote Optical Coherence Tomography in Teleophthalmology in Patients with Eye Disorders-a Systematic Review. Front. Med..

[16] Klein B.E., Klein R. (1982). Cataracts and Macular Degeneration in Older Americans. Arch. Ophthalmol..

[17] Klein R., Klein B.E.K., Linton K.L.P. (1992). Prevalence of Age-Related Maculopathy. The Beaver Dam Eye Study. Ophthalmology.

[18] Congdon N., O’Colmain B., Klaver C.C., Klein R., Muñoz B., Friedman D.S., Kempen J., Taylor H.R., Mitchell P., Eye Diseases Prevalence Research Group (2004). Causes and Prevalence of Visual Impairment among Adults in the United States. Arch. Ophthalmol..

[19] Pascolini D., Mariotti S.P., Pokharel G.P., Pararajasegaram R., Etya’ale D., Négrel A.D., Resnikoff S. (2004). 2002 Global Update of Available Data on Visual Impairment: A Compilation of Population-Based Prevalence Studies. Ophthalmic Epidemiol..

[20] Sasaki M., Kawasaki R., Yanagi Y. (2022). Early Stages of Age-Related Macular Degeneration: Racial/Ethnic Differences and Proposal of a New Classification Incorporating Emerging Concept of Choroidal Pathology. J. Clin. Med..

[21] Ferris F.L., Wilkinson C.P., Bird A., Chakravarthy U., Chew E., Csaky K., Sadda S.R. (2013). Clinical Classification of Age-Related Macular Degeneration. Ophthalmology.

[22] Ambati J., Fowler B.J. (2012). Mechanisms of Age-Related Macular Degeneration. Neuron.

[23] Bird A.C. (2010). Therapeutic Targets in Age-Related Macular Disease. J. Clin. Investig..

[24] Chew E.Y., Clemons T.E., Harrington M., Bressler S.B., Elman M.J., Kim J.E., Garfinkel R., Heier J.S., Brucker A., Boyer D. (2016). Effectiveness of Different Monitoring Modalities in the Detection of Neovascular Age-Related: Macular Degeneration: The HOME Study. Report Number 3. Retina.

[25] Amsler M. (1953). Earliest Symptoms of Diseases of the Macula. Br. J. Ophthalmol..

[26] Trevino R., Kynn M.G. (2008). Macular Function Surveillance Revisited. Optometry.

[27] Schuchard R.A. (1993). Validity and Interpretation of Amsler Grid Reports. Arch. Ophthalmol..

[28] Klein M.L., Ferris F.L., Armstrong J., Hwang T.S., Chew E.Y., Bressler S.B., Chandra S.R. (2008). Retinal Precursors and the Development of Geographic Atrophy in Age-Related Macular Degeneration. Ophthalmology.

[29] Pichi F., Abboud E.B., Ghazi N.G., Khan A.O. (2018). Fundus Autofluorescence Imaging in Hereditary Retinal Diseases. Acta Ophthalmol..

[30] Ly A., Nivison-Smith L., Assaad N., Kalloniatis M. (2016). Infrared Reflectance Imaging in Age-Related Macular Degeneration. Ophthalmic Physiol. Opt..

[31] Joachim N., Colijn J.M., Kifley A., Lee K.E., Buitendijk G.H.S., Klein B.E.K., Myers C.E., Meuer S.M., Tan A.G., Holliday E.G. (2017). Five-Year Progression of Unilateral Age-Related Macular Degeneration to Bilateral Involvement: The Three Continent AMD Consortium Report. Br. J. Ophthalmol..

[32] Lim L.S., Mitchell P., Seddon J.M., Holz F.G., Wong T.Y. (2012). Age-Related Macular Degeneration. Lancet.

[33] Early Treatment Diabetic Retinopathy Study Research Group (1991). Early Treatment Diabetic Retinopathy Study Design and Baseline Patient Characteristics. ETDRS Report Number 7. Ophthalmology.

[34] Lad E.M., Finger R.P., Guymer R. (2023). Biomarkers for the Progression of Intermediate Age-Related Macular Degeneration. Ophthalmol. Ther..

[35] Bhuiyan A., Wong T.Y., Ting D.S.W., Govindaiah A., Souied E.H., Smith R.T. (2020). Artificial Intelligence to Stratify Severity of Age-Related Macular Degeneration (AMD) and Predict Risk of Progression to Late AMD. Transl. Vis. Sci. Technol..

[36] Dow E.R., Jeong H.K., Katz E.A., Toth C.A., Wang D., Lee T., Kuo D., Allingham M.J., Hadziahmetovic M., Mettu P.S. (2023). A Deep-Learning Algorithm to Predict Short-Term Progression to Geographic Atrophy on Spectral-Domain Optical Coherence Tomography. JAMA Ophthalmol..

[37] Wong T.Y., Sun J., Kawasaki R., Ruamviboonsuk P., Gupta N., Lansingh V.C., Maia M., Mathenge W., Moreker S., Muqit M.M.K. (2018). Guidelines on Diabetic Eye Care: The International Council of Ophthalmology Recommendations for Screening, Follow-up, Referral, and Treatment Based on Resource Settings. Ophthalmology.

[38] Haydinger C.D., Ferreira L.B., Williams K.A., Smith J.R. (2023). Mechanisms of Macular Edema. Front. Med..

[39] Horton M.B., Silva P.S., Cavallerano J.D., Aiello L.P. (2016). Clinical Components of Telemedicine Programs for Diabetic Retinopathy. Curr. Diabetes Rep..

[40] Flaxel C.J., Adelman R.A., Bailey S.T., Fawzi A., Lim J.I., Vemulakonda G.A., Ying G.S. (2020). Diabetic Retinopathy Preferred Practice Pattern^®^. Ophthalmology.

[41] Li J.Q., Terheyden J.H., Welchowski T., Schmid M., Letow J., Wolpers C., Holz F.G., Finger R.P. (2019). Prevalence of Retinal Vein Occlusion in Europe: A Systematic Review and Meta-Analysis. Ophthalmologica.

[42] Hayreh S.S., Zimmerman M.B., Podhajsky P. (1994). Incidence of Various Types of Retinal Vein Occlusion and Their Recurrence and Demographic Characteristics. Am. J. Ophthalmol..

[43] Schmidt-Erfurth U., Garcia-Arumi J., Gerendas B.S., Midena E., Sivaprasad S., Tadayoni R., Wolf S., Loewenstein A. (2019). Guidelines for the Management of Retinal Vein Occlusion by the European Society of Retina Specialists (EURETINA). Ophthalmologica.

[44] Schmidt-Erfurth U., Chong V., Loewenstein A., Larsen M., Souied E., Schlingemann R., Eldem B., Monés J., Richard G., Bandello F. (2014). Guidelines for the Management of Neovascular Age-Related Macular Degeneration by the European Society of Retina Specialists (EURETINA). Br. J. Ophthalmol..

[45] ForeseeHome | Home. https://foreseehome.com/.

[46] Mathai M., Reddy S., Elman M.J., Garfinkel R.A., Ladd B., Wagner A.L., Sanborn G.E., Jacobs J.H., Busquets M.A., Chew E.Y. (2022). Analysis of the Long-Term Visual Outcomes of ForeseeHome Remote Telemonitoring: The ALOFT Study. Ophthalmol. Retin..

[47] Loewenstein A., Ferencz J.R., Lang Y., Yeshurun I., Pollack A., Siegal R., Lifshitz T., Karp J., Roth D., Bronner G. (2010). Toward Earlier Detection of Choroidal Neovascularization Secondary to Age-Related Macular Degeneration: Multicenter Evaluation of a Preferential Hyperacuity Perimeter Designed as a Home Device. Retina.

[48] Chew E.Y., Clemons T.E., Bressler S.B., Elman M.J., Danis R.P., Domalpally A., Heier J.S., Kim J.E., Garfinkel R. (2014). Randomized Trial of a Home Monitoring System for Early Detection of Choroidal Neovascularization Home Monitoring of the Eye (HOME) Study. Ophthalmology.

[49] Nahen K., Benyamini G., Loewenstein A. (2020). Evaluation of a Self-Imaging SD-OCT System for Remote Monitoring of Patients with Neovascular Age Related Macular Degeneration. Klin. Monatsblatter Augenheilkd..

[50] Keenan T.D.L., Goldstein M., Goldenberg D., Zur D., Shulman S., Loewenstein A. (2021). Prospective, Longitudinal Pilot Study: Daily Self-Imaging with Patient-Operated Home OCT in Neovascular Age-Related Macular Degeneration. Ophthalmol. Sci..

[51] Von Der Burchard C., Sudkamp H., Tode J., Ehlken C., Purtskhvanidze K., Moltmann M., Heimes B., Koch P., Münst M., Vom Endt M. (2022). Self-Examination Low-Cost Full-Field Optical Coherence Tomography (SELFF-OCT) for Neovascular Age-Related Macular Degeneration: A Cross-Sectional Diagnostic Accuracy Study. BMJ Open.

[52] Olshausen B.A., Field D.J. (1996). Emergence of Simple-Cell Receptive Field Properties by Learning a Sparse Code for Natural Images. Nature.

[53] Liu X., Kang J.U. (2011). Sparse OCT: Optimizing Compressed Sensing in Spectral Domain Optical Coherence Tomography. Proc. SPIE.

[54] Maloca P., Hasler P.W., Barthelmes D., Arnold P., Matthias M., Scholl H.P.N., Gerding H., Garweg J., Heeren T., Balaskas K. (2018). Safety and Feasibility of a Novel Sparse Optical Coherence Tomography Device for Patient-Delivered Retina Home Monitoring. Transl. Vis. Sci. Technol..

[55] Malone J.D., Hussain I., Bowden A.K. (2023). SmartOCT: Smartphone-Integrated Optical Coherence Tomography. Biomed. Opt. Express.

[56] Luu K.T., Seal J., Green M., Winskill C., Attar M. (2022). Effect of Anti-VEGF Therapy on the Disease Progression of Neovascular Age-Related Macular Degeneration: A Systematic Review and Model-Based Meta-Analysis. J. Clin. Pharmacol..

[57] Bakri S.J., Thorne J.E., Ho A.C., Ehlers J.P., Schoenberger S.D., Yeh S., Kim S.J. (2019). Safety and Efficacy of Anti-Vascular Endothelial Growth Factor Therapies for Neovascular Age-Related Macular Degeneration: A Report by the American Academy of Ophthalmology. Ophthalmology.

[58] Wykoff C.C., Clark W.L., Nielsen J.S., Brill J.V., Greene L.S., Heggen C.L. (2018). Optimizing Anti-VEGF Treatment Outcomes for Patients with Neovascular Age-Related Macular Degeneration. J. Manag. Care Spec. Pharm..

[59] Almony A., Keyloun K.R., Shah-Manek B., Multani J.K., McGuiness C.B., Chen C.C., Campbell J.H. (2021). Clinical and Economic Burden of Neovascular Age-Related Macular Degeneration by Disease Status: A US Claims-Based Analysis. J. Manag. Care Spec. Pharm..

[60] Notal Vision. https://notalvision.com/.

[61] Holekamp N.M., de Beus A.M., Clark W.L., Heier J.S. (2024). Prospective trial of home optical coherence tomography-guided management of treatment experienced neovascular age-related macular degeneration patients. Retina.

[62] Seong D., Han S., Kang D., Najnin T., Saleah S.A., Gi W., Jeon M., Kim J. (2024). Development of Single-Board Computer-Based Temperature-Insensitive Compact Optical Coherence Tomography for Versatile Applications. IEEE Trans. Instrum. Meas..

[63] Rank E.A., Agneter A., Schmoll T., Leitgeb R.A., Drexler W. (2022). Miniaturizing Optical Coherence Tomography. Transl. Biophotonics.

[64] Duan Z., Huang K., Luo Z., Ma K., Wang G., Hu X., Zhang J., Luo X., Huang Y., Liu G. (2022). Portable Boom-Type Ultrahigh-Resolution OCT with an Integrated Imaging Probe for Supine Position Retinal Imaging. Biomed. Opt. Express.

[65] Nakamura M., Hirano T., Chiku Y., Takahashi Y., Miyasaka H., Kakihara S., Hoshiyama K., Murata T. (2024). Reproducibility of Portable OCT and Comparison with Conventional OCT. Diagnostics.

[66] Grulkowski I., Liu J.J., Potsaid B., Jayaraman V., Lu C.D., Jiang J., Cable A.E., Duker J.S., Fujimoto J.G. (2012). Retinal, Anterior Segment and Full Eye Imaging Using Ultrahigh Speed Swept Source OCT with Vertical-Cavity Surface Emitting Lasers. Biomed. Opt. Express.

[67] Maamari R.N., Keenan J.D., Fletcher D.A., Margolis T.P. (2014). A Mobile Phone-Based Retinal Camera for Portable Wide Field Imaging. Br. J. Ophthalmol..

[68] Fang H.S., Bai C.H., Cheng C.K. (2023). Strict Pro Re Nata versus treat-and-extend regimens in neovascular age-related macular degeneration: A systematic review and meta-analysis. Retina.

[69] Fung A.E., Lalwani G.A., Rosenfeld P.J., Dubovy S.R., Michels S., Feuer W.J., Puliafito C.A., Davis J.L., Flynn H.W., Esquiabro M. (2007). An Optical Coherence Tomography-Guided, Variable Dosing Regimen with Intravitreal Ranibizumab (Lucentis) for Neovascular Age-Related Macular Degeneration. Am. J. Ophthalmol..

[70] Heier J.S., Liu Y., Holekamp N.M., Ali M.H., Astafurov K., Blinder K.J., Busquets M.A., Chica M.A., Elman M.J., Fein J.G. (2024). Clinical Use of Home OCT Data to Manage Neovascular Age-Related Macular Degeneration. J. Vitreoretin. Dis..

[71] Islam R., Patamsetti V., Gadhi A., Gondu R.M., Bandaru C.M., Kesani S.C., Abiona O., Islam R., Patamsetti V., Gadhi A. (2023). The Future of Cloud Computing: Benefits and Challenges. Int. J. Commun. Netw. Syst. Sci..

[72] Kim J.E., Tomkins-Netzer O., Elman M.J., Lally D.R., Goldstein M., Goldenberg D., Shulman S., Benyamini G., Loewenstein A. (2022). Evaluation of a Self-Imaging SD-OCT System Designed for Remote Home Monitoring. BMC Ophthalmol..

[73] Yu H.J., Kiernan D.F., Eichenbaum D., Sheth V.S., Wykoff C.C. (2021). Home Monitoring of Age-Related Macular Degeneration: Utility of the ForeseeHome Device for Detection of Neovascularization. Ophthalmol. Retin..

[74] Woodward M.A., Ple-Plakon P., Blachley T., Musch D.C., Newman-Casey P.A., De Lott L.B., Lee P.P. (2015). Eye Care Providers’ Attitudes towards Tele-Ophthalmology. Telemed. J. E Health.

[75] Bajra R., Srinivasan M., Torres E.C., Rydel T., Schillinger E. (2023). Training Future Clinicians in Telehealth Competencies: Outcomes of a Telehealth Curriculum and TeleOSCEs at an Academic Medical Center. Front. Med..

[76] Ben Rebah H., Ben Sta H. (2018). Cloud Computing: Potential Risks and Security Approaches. e-Infrastructure and e-Services for Developing Countries.

[77] Grande D., Luna Marti X., Feuerstein-Simon R., Merchant R.M., Asch D.A., Lewson A., Cannuscio C.C. (2020). Health Policy and Privacy Challenges Associated With Digital Technology. JAMA Netw. Open.

[78] Mansberger S.L., Sheppler C., Barker G., Gardiner S.K., Demirel S., Wooten K., Becker T.M. (2015). Long-Term Comparative Effectiveness of Telemedicine in Providing Diabetic Retinopathy Screening Examinations: A Randomized Clinical Trial. JAMA Ophthalmol..

[79] Sasongko M.B., Indrayanti S.R., Wardhana F.S., Widhasari I.A., Widyaputri F., Prayoga M.E., Widayanti T.W., Supanji, Agni A.N. (2021). Low Utility of Diabetic Eye Care Services and Perceived Barriers to Optimal Diabetic Retinopathy Management in Indonesian Adults with Vision-Threatening Diabetic Retinopathy. Diabetes Res. Clin. Pract..

[80] Lestari Y.D., Adriono G.A., Ratmilia R., Magdalena C., Sitompul R. (2023). Knowledge, Attitude, and Practice Pattern towards Diabetic Retinopathy Screening among General Practitioners in Primary Health Centres in Jakarta, the Capital of Indonesia. BMC Prim. Care.

[81] Lee S.C., Lieng M.K., Alber S., Mehta N., Emami-Naeini P., Yiu G. (2022). Trends in Remote Retinal Imaging Utilization and Payments in the United States. Ophthalmology.

[82] Hernandez R., Kennedy C., Banister K., Goulao B., Cook J., Sivaprasad S., Hogg R., Azuara-Blanco A., Heimann H., Dimitrova M. (2022). Early Detection of Neovascular Age-Related Macular Degeneration: An Economic Evaluation Based on Data from the EDNA Study. Br. J. Ophthalmol..

[83] Ecsedy M., Szamosi A., Karkó C., Zubovics L., Varsányi B., Németh J., Récsán Z. (2007). A Comparison of Macular Structure Imaged by Optical Coherence Tomography in Preterm and Full-Term Children. Investig. Ophthalmol. Vis. Sci..

[84] Skarmoutsos F., Sandhu S.S., Voros G.M., Shafiq A. (2006). The Use of Optical Coherence Tomography in the Management of Cystoid Macular Edema in Pediatric Uveitis. J. AAPOS.

[85] Patel C.K. (2006). Optical Coherence Tomography in the Management of Acute Retinopathy of Prematurity. Am. J. Ophthalmol..

[86] Ehlers J.P. (2016). Intraoperative Optical Coherence Tomography: Past, Present, and Future. Eye.

[87] Ehlers J.P., Dupps W.J., Kaiser P.K., Goshe J., Singh R.P., Petkovsek D., Srivastava S.K. (2014). The Prospective Intraoperative and Perioperative Ophthalmic ImagiNg with Optical CoherEncE TomogRaphy (PIONEER) Study: 2-Year Results. Am. J. Ophthalmol..

[88] Ehlers J.P., Kaiser P.K., Srivastava S.K. (2014). Intraoperative Optical Coherence Tomography Using the RESCAN 700: Preliminary Results from the DISCOVER Study. Br. J. Ophthalmol..

[89] Ehlers J.P., Srivastava S.K., Feiler D., Noonan A.I., Rollins A.M., Tao Y.K. (2014). Integrative Advances for OCT-Guided Ophthalmic Surgery and Intraoperative OCT: Microscope Integration, Surgical Instrumentation, and Heads-up Display Surgeon Feedback. PLoS ONE.

[90] ZEISS OPMI LUMERA 700 Ophthalmic Microscope. https://www.zeiss.com/meditec/en/products/surgical-microscopes/ophthalmic-microscopes/opmi-lumera-700.html.

[91] EnFocus Intraoperative OCT Imaging System | Products | Leica Microsystems. https://www.leica-microsystems.com/products/surgical-microscopes/p/enfocus/.

[92] OphthalmologyHaag-Streit Far East | Haag-Streit. http://www.hs-fe.com/haag-streit-surgical/ophthalmology/ioct.

[93] Browne A.W., Ehlers J.P., Sharma S., Srivastava S.K. (2017). Intraoperative OCT-Assisted Chorioretinal Biopsy in the DISCOVER Study. Retina.

[94] Ehlers J.P., Uchida A., Srivastava S.K. (2017). Intraoperative Optical Coherence Tomography-Compatible Surgical Instruments for Real-Time Image-Guided Ophthalmic Surgery. Br. J. Ophthalmol..

[95] Ehlers J.P., Modi Y.S., Pecen P.E., Goshe J., Dupps W.J., Rachitskaya A., Sharma S., Yuan A., Singh R., Kaiser P.K. (2018). The DISCOVER Study 3-Year Results: Feasibility and Usefulness of Microscope-Integrated Intraoperative OCT during Ophthalmic Surgery. Ophthalmology.

[96] Lu C.D., Waheed N.K., Witkin A., Baumal C.R., Liu J.J., Potsaid B., Joseph A., Jayaraman V., Cable A., Chan K. (2018). Microscope-Integrated Intraoperative Ultrahigh-Speed Swept-Source Optical Coherence Tomography for Widefield Retinal and Anterior Segment Imaging. Ophthalmic Surg. Lasers Imaging Retin..

[97] Gabr H., Chen X., Zevallos-Carrasco O.M., Viehland C., Dandrige A., Sarin N., Mahmoud T.H., Vajzovic L., Izatt J.A., Toth C.A. (2018). Visualization from intraoperative swept-source microscope-integrated optical coherence tomography in vitrectomy for complications of proliferative diabetic retinopathy. Retina.

[98] Grewal D.S., Carrasco-Zevallos O.M., Gunther R., Izatt J.A., Toth C.A., Hahn P. (2017). Intra-Operative Microscope-Integrated Swept-Source Optical Coherence Tomography Guided Placement of Argus II Retinal Prosthesis. Acta Ophthalmol..

[99] Mura M., Barca F. (2014). Intraocular Optical Coherence Tomography. Dev. Ophthalmol..

[100] Song C., Gehlbach P.L., Kang J.U., Taylor R., Jensen P., Whitcomb L., Barnes A., Kumar R., Stoianovici D., Gupta P. (2012). Active Tremor Cancellation by a “Smart” Handheld Vitreoretinal Microsurgical Tool Using Swept Source Optical Coherence Tomography. Opt. Express.

[101] Liang C.-P., Wierwille J., Moreira T., Schwartzbauer G., Jafri M.S., Tang C.-M., Chen Y. (2011). A Forward-Imaging Needle-Type OCT Probe for Image Guided Stereotactic Procedures. Opt. Express.

[102] Yenerel N.M., Kucumen R.B., Gorgun E. (2013). The Complementary Benefit of Anterior Segment Optical Coherence Tomography in Penetrating Keratoplasty. Clin. Ophthalmol..

[103] Mimouni M., Kronschläger M., Ruiss M., Findl O. (2021). Intraoperative Optical Coherence Tomography Guided Corneal Sweeping for Removal of Remnant Interface Fluid during Ultra-Thin Descemet Stripping Automated Endothelial Keratoplasty. BMC Ophthalmol..

[104] Steverink J.G., Wisse R.P.L. (2017). Intraoperative Optical Coherence Tomography in Descemet Stripping Automated Endothelial Keratoplasty: Pilot Experiences. Int. Ophthalmol..

[105] Muijzer M.B., Schellekens P.A.W.J., Beckers H.J.M., de Boer J.H., Imhof S.M., Wisse R.P.L. (2022). Clinical Applications for Intraoperative Optical Coherence Tomography: A Systematic Review. Eye.

[106] Rechichi M., Mazzotta C., Daya S., Mencucci R., Lanza M., Meduri A. (2016). Intraoperative OCT Pachymetry in Patients Undergoing Dextran-Free Riboflavin UVA Accelerated Corneal Collagen Crosslinking. Curr. Eye Res..

[107] Siebelmann S., Horstmann J., Scholz P., Bachmann B., Matthaei M., Hermann M., Cursiefen C. (2018). Intraoperative Changes in Corneal Structure during Excimer Laser Phototherapeutic Keratectomy (PTK) Assessed by Intraoperative Optical Coherence Tomography. Graefes Arch. Clin. Exp. Ophthalmol..

[108] Gozawa M., Takamura Y., Miyake S., Yokota S., Sakashita M., Arimura S., Takihara Y., Inatani M. (2016). Prospective Observational Study of Conjunctival Scarring after Phacoemulsification. Acta Ophthalmol..

[109] Banayan N., Georgeon C., Grieve K., Borderie V.M. (2018). Spectral-Domain Optical Coherence Tomography in Limbal Stem Cell Deficiency. A Case-Control Study. Am. J. Ophthalmol..

[110] Muijzer M.B., Soeters N., Godefrooij D.A., Van Luijk C.M., Wisse R.P.L. (2020). Intraoperative Optical Coherence Tomography-Assisted Descemet Membrane Endothelial Keratoplasty: Toward More Efficient, Safer Surgery. Cornea.

[111] Sharma N., Singhal D., Maharana P.K., Jain R., Sahay P., Titiyal J.S. (2018). Continuous Intraoperative Optical Coherence Tomography-Guided Shield Ulcer Debridement with Tuck in Multilayered Amniotic Membrane Transplantation. Indian J. Ophthalmol..

[112] Palanker D.V., Blumenkranz M.S., Andersen D., Wiltberger M., Marcellino G., Gooding P., Angeley D., Schuele G., Woodley B., Simoneau M. (2010). Femtosecond Laser-Assisted Cataract Surgery with Integrated Optical Coherence Tomography. Sci. Transl. Med..

[113] Salgado R., Torres P., Marinho A. (2024). Update on Femtosecond Laser-Assisted Cataract Surgery: A Review. Clin. Ophthalmol..

[114] Titiyal J.S. (2024). Imaging in Cataract Surgery. Indian J. Ophthalmol..

[115] Khoramnia R., Auffarth G., Łabuz G., Pettit G., Suryakumar R. (2022). Refractive Outcomes after Cataract Surgery. Diagnostics.

[116] Kan J.T.C., Betzler B.K., Lim S.Y., Ang B.C.H. (2022). Anterior Segment Imaging in Minimally Invasive Glaucoma Surgery—A Systematic Review. Acta Ophthalmol..

[117] Kudsieh B., Fernández-Vigo J.I., Canut Jordana M.I., Vila-Arteaga J., Urcola J.A., Ruiz Moreno J.M., García-Feijóo J., Fernández-Vigo J.Á. (2022). Updates on the Utility of Anterior Segment Optical Coherence Tomography in the Assessment of Filtration Blebs after Glaucoma Surgery. Acta Ophthalmol..

[118] Jung K.I., Lim S.A., Park H.Y.L., Park C.K. (2013). Visualization of Blebs Using Anterior-Segment Optical Coherence Tomography after Glaucoma Drainage Implant Surgery. Ophthalmology.

[119] Tan J., Roney M., Choudhary A., Batterbury M., Vallabh N.A. (2024). Visualization of Scleral Flap Patency in Glaucoma Filtering Blebs Using OCT. Ophthalmol. Sci..

[120] Mastropasqua R., Fasanella V., Agnifili L., Curcio C., Ciancaglini M., Mastropasqua L. (2014). Anterior Segment Optical Coherence Tomography Imaging of Conjunctival Filtering Blebs after Glaucoma Surgery. BioMed Res. Int..

[121] Eha J., Hoffmann E.M., Pfeiffer N. (2013). Long-Term Results after Transconjunctival Resuturing of the Scleral Flap in Hypotony Following Trabeculectomy. Am. J. Ophthalmol..

[122] Kaur S., Bradfield Y., AS V., Gupta K., Gupta P., Sukhija J. (2024). Anterior Segment Optical Coherence Tomography (AS-OCT) in Strabismus Following Trauma. J. AAPOS.

[123] Gupta N., Varshney A., Ramappa M., Basu S., Romano V., Acharya M., Gaur A., Kapur N., Singh A., Shah G. (2022). Role of AS-OCT in Managing Corneal Disorders. Diagnostics.

[124] Naujokaitis T., Auffarth G.U., Łabuz G., Kessler L.J., Khoramnia R. (2023). Diagnostic Techniques to Increase the Safety of Phakic Intraocular Lenses. Diagnostics.

[125] Urkude J., Titiyal J.S., Sharma N. (2017). Intraoperative Optical Coherence Tomography-Guided Management of Cap-Lenticule Adhesion During SMILE. J. Refract. Surg..

[126] Rosas Salaroli C.H., Li Y., Huang D. (2009). High-Resolution Optical Coherence Tomography Visualization of LASIK Flap Displacement. J. Cataract. Refract. Surg..

[127] Iovieno A., Sharma D.P., Wilkins M.R. (2012). OCT Visualization of Corneal Structural Changes in Traumatic Dislocation of LASIK Flap. Int. Ophthalmol..

[128] Dhaini A.R., Fattah M.A., El-Oud S.M., Awwad S.T. (2018). Automated Detection and Classification of Corneal Haze Using Optical Coherence Tomography in Patients With Keratoconus After Cross-Linking. Cornea.

[129] Hafezi F., Lu N.J., Assaf J.F., Hafezi N.L., Koppen C., Vinciguerra R., Vinciguerra P., Hillen M., Awwad S.T. (2022). Demarcation Line Depth in Epithelium-Off Corneal Cross-Linking Performed at the Slit Lamp. J. Clin. Med..

[130] Ehlers J.P., Goshe J., Dupps W.J., Kaiser P.K., Singh R.P., Gans R., Eisengart J., Srivastava S.K. (2015). Determination of Feasibility and Utility of Microscope-Integrated OCT During Ophthalmic Surgery: The DISCOVER Study RESCAN Results. JAMA Ophthalmol..

[131] Fung A.T., Galvin J., Tran T. (2021). Epiretinal Membrane: A Review. Clin. Exp. Ophthalmol..

[132] Ehlers J.P., Kernstine K., Farsiu S., Sarin N., Maldonado R., Toth C.A. (2011). Analysis of Pars Plana Vitrectomy for Optic Pit–Related Maculopathy With Intraoperative Optical Coherence Tomography: A Possible Connection With the Vitreous Cavity. Arch. Ophthalmol..

[133] Ehlers J.P., McNutt S., Dar S., Tao Y.K., Srivastava S.K. (2014). Visualisation of Contrast-Enhanced Intraoperative Optical Coherence Tomography with Indocyanine Green. Br. J. Ophthalmol..

[134] Zhang J., Zhang C., Xie H., Luo D., Zhang J. (2024). Intravitreal Indocyanine Green Is Toxic to the Retinal Cells. Biochem. Biophys. Res. Commun..

[135] Ando F., Yasui O., Hirose H., Ohba N. (2004). Optic Nerve Atrophy after Vitrectomy with Indocyanine Green-Assisted Internal Limiting Membrane Peeling in Diffuse Diabetic Macular Edema. Adverse Effect of ICG-Assisted ILM Peeling. Graefes Arch. Clin. Exp. Ophthalmol..

[136] Gandorfer A., Haritoglou C., Kampik A. (2008). Toxicity of Indocyanine Green in Vitreoretinal Surgery. Dev. Ophthalmol..

[137] Falkner-Radler C.I., Glittenberg C., Gabriel M., Binder S. (2015). Intrasurgical microscope-integrated spectral domain optical coherence tomography-assisted membrane peeling. Retina.

[138] Leisser C., Hackl C., Hirnschall N., Luft N., Döller B., Draschl P., Rigal K., Findl O. (2016). Visualizing Macular Structures During Membrane Peeling Surgery With an Intraoperative Spectral-Domain Optical Coherence Tomography Device. Ophthalmic Surg. Lasers Imaging Retin..

[139] Bikbova G., Oshitari T., Baba T., Yamamoto S., Mori K. (2019). Pathogenesis and Management of Macular Hole: Review of Current Advances. J. Ophthalmol..

[140] Gaudric A., Haouchine B., Massin P., Paques M., Blain P., Erginay A. (1999). Macular Hole Formation: New Data Provided by Optical Coherence Tomography. Arch. Ophthalmol..

[141] Abdul-Kadir M.A., Lim L.T. (2021). Update on Surgical Management of Complex Macular Holes: A Review. Int. J. Retin. Vitr..

[142] Ehlers J.P., Xu D., Kaiser P.K., Singh R.P., Srivastava S.K. (2014). Intrasurgical Dynamics of Macular Hole Surgery: An Assessment of Surgery-Induced Ultrastructural Alterations with Intraoperative Optical Coherence Tomography. Retina.

[143] Ehlers J.P., Uchida A., Srivastava S.K., Hu M. (2019). Predictive Model for Macular Hole Closure Speed: Insights From Intraoperative Optical Coherence Tomography. Transl. Vis. Sci. Technol..

[144] Kumar V., Yadav B. (2018). HOLE-DOOR SIGN: A Novel Intraoperative Optical Coherence Tomography Feature Predicting Macular Hole Closure. Retina.

[145] Lorusso M., Micelli Ferrari L., Cicinelli M.V., Nikolopoulou E., Zito R., Bandello F., Querques G., Micelli Ferrari T. (2020). Feasibility and Safety of Intraoperative Optical Coherence Tomography-Guided Short-Term Posturing Prescription after Macular Hole Surgery. Ophthalmic Res..

[146] Allen A., Zheng Y., Lee T., Joseph S., Zhang X., Feng H.L., Fekrat S. (2024). Risk Factors for Progression of Vitreomacular Traction to Macular Hole. J. Vitreoretin. Dis..

[147] Morescalchi F., Russo A., Semeraro F. (2021). Surgical outcomes of vitreomacular traction treated with foveal-sparing peeling of the internal limiting membrane. Retina.

[148] Abraham J.R., Srivastava S.K., K Le T., Sharma S., Rachitskaya A., Reese J.L., Ehlers J.P. (2020). Intraoperative OCT-Assisted Retinal Detachment Repair in the DISCOVER Study: Impact and Outcomes. Ophthalmol. Retin..

[149] Heydinger S., Wang A.L., Ufret-Vincenty R., Robertson Z.M., He Y.G. (2023). Comparison of Surgical Outcomes for Uncomplicated Primary Retinal Detachment Repair. Clin. Ophthalmol..

[150] Lee L.B., Srivastava S.K. (2011). Intraoperative Spectral-Domain Optical Coherence Tomography during Complex Retinal Detachment Repair. Ophthalmic Surg. Lasers Imaging.

[151] Toygar O., Riemann C.D. (2016). Intraoperative Optical Coherence Tomography in Macula Involving Rhegmatogenous Retinal Detachment Repair with Pars Plana Vitrectomy and Perfluoron. Eye.

[152] Ehlers J.P., Ohr M.P., Kaiser P.K., Srivastava S.K. (2013). Novel Microarchitectural Dynamics in Rhegmatogenous Retinal Detachments Identified with Intraoperative Optical Coherence Tomography. Retina.

[153] Eckardt F., Klaas J., Siedlecki J., Schworm B., Keidel L.F., Vogt D., Kreutzer T., Priglinger S. (2025). Internal Limiting Membrane Peeling in Primary Rhegmatogenous Retinal Detachment: Functional and Morphologic Results. Klin. Monatsblatter Augenheilkd..

[154] Gregori N.Z., Lam B.L., Davis J.L. (2019). Intraoperative Use of Microscope-Integrated Optical Coherence Tomography for Subretinal Gene Therapy Delivery. Retina.

[155] Vajzovic L., Sleiman K., Viehland C., Carrasco-Zevallos O.M., Klingeborn M., Dandridge A., Bowes Rickman C., Izatt J.A., Toth C.A. (2019). Four-Dimensional Microscope-Integrated Optical Coherence Tomography Guidance in a Model Eye Subretinal Surgery. Retina.

[156] Hussain R.M., Tran K.D., Berrocal A.M., Maguire A.M. (2019). Subretinal Injection of Voretigene Neparvovec-Rzyl in a Patient With RPE65-Associated Leber’s Congenital Amaurosis. Ophthalmic Surg. Lasers Imaging Retin..

[157] Vasconcelos H.M., Lujan B.J., Pennesi M.E., Yang P., Lauer A.K. (2020). Intraoperative Optical Coherence Tomographic Findings in Patients Undergoing Subretinal Gene Therapy Surgery. Int. J. Retin. Vitr..

[158] Ehlers J.P., Petkovsek D.S., Yuan A., Singh R.P., Srivastava S.K. (2015). Intrasurgical Assessment of Subretinal TPA Injection for Submacular Hemorrhage in the PIONEER Study Utilizing Intraoperative OCT. Ophthalmic Surg. Lasers Imaging Retin..

[159] Smith A.G., Cost B.M., Ehlers J.P. (2015). Intraoperative OCT-Assisted Subretinal Perfluorocarbon Liquid Removal in the DISCOVER Study. Ophthalmic Surg. Lasers Imaging Retin..

[160] Valikodath N.G., Li J.D., Raynor W., Izatt J.A., Toth C.A., Vajzovic L. (2024). Intraoperative OCT-Guided Volumetric Measurements of Subretinal Therapy Delivery in Humans. J. Vitreoretin. Dis..

[161] Gan N., Lam W.-C. (2018). Special Considerations for Pediatric Vitreoretinal Surgery. Taiwan J. Ophthalmol..

[162] Maldonado R.S., Izatt J.A., Sarin N., Wallace D.K., Freedman S., Cotten C.M., Toth C.A. (2010). Optimizing Hand-Held Spectral Domain Optical Coherence Tomography Imaging for Neonates, Infants, and Children. Investig. Ophthalmol. Vis. Sci..

[163] Lee H., Purohit R., Patel A., Papageorgiou E., Sheth V., Maconachie G., Pilat A., McLean R.J., Proudlock F.A., Ottlob I. (2015). In Vivo Foveal Development Using Optical Coherence Tomography. Investig. Ophthalmol. Vis. Sci..

[164] Chavala S.H., Farsiu S., Maldonado R., Wallace D.K., Freedman S.F., Toth C.A. (2009). Insights into Advanced Retinopathy of Prematurity Using Handheld Spectral Domain Optical Coherence Tomography Imaging. Ophthalmology.

[165] Vinekar A., Sivakumar M., Shetty R., Mahendradas P., Krishnan N., Mallipatna A., Shetty K.B. (2010). A Novel Technique Using Spectral-Domain Optical Coherence Tomography (Spectralis, SD-OCT+HRA) to Image Supine Non-Anaesthetized Infants: Utility Demonstrated in Aggressive Posterior Retinopathy of Prematurity. Eye.

[166] Cao C., Markovitz M., Ferenczy S., Shields C.L. (2014). Hand-Held Spectral-Domain Optical Coherence Tomography of Small Macular Retinoblastoma in Infants before and after Chemotherapy. J. Pediatr. Ophthalmol. Strabismus.

[167] Malik K., Welch R.J., Shields C.L. (2020). Hand-held optical coherence tomography monitoring of chemoresistant retinoblastoma. Retin. Cases Brief. Rep..

[168] Rothman A.L., Folgar F.A., Tong A.Y., Toth C.A. (2014). Spectral Domain Optical Coherence Tomography Characterization of Pediatric Epiretinal Membranes. Retina.

[169] Chen X., Viehland C., Carrasco-Zevallos O.M., Keller B., Vajzovic L., Izatt J.A., Toth C.A. (2017). Microscope-Integrated Optical Coherence Tomography Angiography in the Operating Room in Young Children With Retinal Vascular Disease. JAMA Ophthalmol..

[170] da Cruz N.F.S., Sengillo J.D., Hudson J.L., Carletti P., de Oliveira G., Negron C.I., Felder M.B., Berrocal A.M. (2023). Intraoperative OCT Angiography in Pediatric Patients with Persistent Fetal Vasculature. Ophthalmol. Retin..

[171] Juergens L., Michiels S., Borrelli M., Spaniol K., Guthoff R., Schrader S., Frings A., Geerling G. (2021). Intraoperative OCT-Real-World User Evaluation in Routine Surgery. Klin. Monatsblatter Augenheilkd..

[172] van der Zee C., Muijzer M.B., van den Biggelaar F.J.H.M., Nuijts R.M.M.A., Delbeke H., Dickman M.M., Imhof S.M., Wisse R.P.L. (2024). Cost-Effectiveness of the ADVISE Trial: An Intraoperative OCT Protocol in DMEK Surgery. Acta Ophthalmol..

[173] Zakir R., Iqbal K., Ali M.H., Mirza U.T., Mahmood K., Riaz S., Hashmani N. (2022). The Outcomes and Usefulness of Intraoperative Optical Coherence Tomography in Vitreoretinal Surgery and Its Impact on Surgical Decision Making. Rom. J. Ophthalmol..

